# Understanding Metal–Organic Framework Densification: Solvent Effects and the Growth of Colloidal Primary Nanoparticles in Monolithic ZIF‐8

**DOI:** 10.1002/smll.202500510

**Published:** 2025-04-13

**Authors:** Ayush Pathak, Lana A. Alghamdi, Javier Fernández‐Catalá, Michele Tricarico, Diego Cazorla‐Amorós, Jin‐Chong Tan, Ángel Berenguer‐Murcia, Gift Mehlana, Andrew E. H. Wheatley

**Affiliations:** ^1^ Yusuf Hamied Department of Chemistry University of Cambridge Lensfield Road Cambridge CB2 1EW UK; ^2^ Centre of Excellence for Nanomaterials for Clean Energy Applications King Abdulaziz City for Science and Technology P.O. Box 6086 Riyadh 11442 Saudi Arabia; ^3^ Department of Inorganic Chemistry and Materials Institute Universidad de Alicante Alicante Apdo. 99 Spain; ^4^ Multifunctional Materials & Composites (MMC) Laboratory, Department of Engineering Science University of Oxford Parks Road Oxford OX1 3PJ UK; ^5^ Department of Chemical Sciences Faculty of Science and Technology Midlands State University P Bag 9055, Senga Road Gweru Zimbabwe

**Keywords:** metal–organic frameworks, monolith, sol–gel synthesis, solvent effects, ZIF‐8

## Abstract

To commercialize metal–organic frameworks (MOFs), it is vital they are made easier to handle. There have been many attempts to synthesize them as pellets, tablets, or granules, though they come with innate drawbacks. Only recently have these been overcome, through the advent of self‐shaping densified or monolithic MOFs (_mono_MOFs), which require minimal post‐synthetic modification and avoid poor structural integrity, intractability, and pore collapse or blockage. ZIF‐8 (zeolitic imidazolate framework‐8) has emerged as a prototypical _mono_MOF in pure and in situ doped forms. Now its formation in solvent mixtures is studied to better understand the early stages of monolith formation and improve the scope of monoliths for hosting solvent‐sensitive guests. Solvent‐, temperature‐ and coagulant‐dependent control over reaction kinetics induces variations in morphology that are explained by relating the nucleation and growth rates of primary nanocrystallites to the stability of colloidal dispersions during reaction. This yields mesoporous _mono_ZIF‐8 with mean pore size 16 nm, S_BET_ >1400 m^2 ^g^−1^, bulk density 0.76 g cm^−3^, and resistance to permanent deformation exceeding previous reports. While the study highlights the powerful manipulation of _mono_MOF characteristics, a new understanding of the growth and stability of primary nanocrystallites has consequences for colloid synthesis generally.

## Graphical abstract

Solvent effects are harnessed to manipulate the rate of nucleation of primary nanocrystallites in prototypical MOF ZIF‐8. Aggregation is fine‐tuned by modifying reaction temperature and by applying DLVO theory, shedding light on the emerging field of conformed MOF synthesis and promoting their use with biological systems.

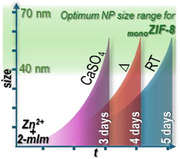

## 1 Introduction

Reticular chemistry has emerged as a powerful tool for articulating crystalline materials promising varied applications. The rapid development of the field has been enabled by the topological diversity of the constituent simple chemical building blocks.^[^ ^1^ ^]^ Metal–organic frameworks (MOFs)^[^ ^2^ ^]^ represent prototypical reticular frameworks^[^ ^3^ ^]^ and are notable for their ease of self‐assembly, structural tunability, multifunctionality, the scope for post‐synthetic modification, and, in many cases, porosity and high surface area.^[^ ^4^ ^]^ This combination of properties makes them exceptional candidates for applications in fields that include catalysis, gas storage, biomedicine, water treatment, and molecular recognition.^[^ ^5^ ^]^ However, with only a very few exceptions,^[^ ^6^ ^]^ the commercialization of MOFs has proved problematic due to the inconsistent nature of the polydisperse microcrystalline powders they tend to form.^[^ ^5^, ^7^ ^]^ This has several consequences. First, during normal usage, MOFs can suffer slow compaction (e.g., under flow conditions) which causes pressure drops and resistance to gas flow. Powder MOFs also exhibit difficulties in handling, are mechanically fragile, and are susceptible to pore collapse and blockage during use.^[^ ^7^, ^8^ ^]^ Moreover, in applications such as gas storage, volumetric capacities have been frequently obtained by converting gravimetric uptake assuming the MOF to exhibit its ideal single‐crystal density and overlooking the extensive interparticle porosity synonymous with powder formation.^[^ ^9^ ^]^ The practical consequence of this is that the adsorbent volumetric capacity is significantly reduced,^[^ ^10^ ^]^ with even experimentally compacted MOF pellets potentially exhibiting bulk densities significantly less than single crystal density. Also, in its powder form, a MOF can be difficult to handle, suffering dusting and material loss,^[^ ^8a^ ^]^ as well as being energy‐intensive and potentially inefficient to recycle (by centrifugation), all of which can have major cost and environmental implications.^[^ ^7^, ^8^ ^]^

Efforts to overcome the drawbacks outlined above led to the development of processed MOFs.^[^ ^11^ ^]^ These were materials subjected to mechanical pressing (MOF is compressed in a mold with or without binding agents), substrate coating, foaming (gas is introduced into a particulate MOF suspension and then dried), or granulation (binding agents and active MOFs are mixed to form granules).^[^ ^8^, ^12^ ^]^ However, while they address the powdery consistency of the MOF, they have tended to introduce other drawbacks. Hence, while pressing raises mechanical stability^[^ ^13^ ^]^ it can cause catastrophic framework collapse through either amorphization resulting from covalent bond cleavage^[^ ^7a^ ^]^ or phase transitions.^[^ ^14^ ^]^ Meanwhile, binding agent(s) introduce additional functionality, add synthetic complexity, and tend to induce pore blockage whilst failing to necessarily incur a significant increase in density.^[^ ^15^ ^]^ Overall, any advances in mechanical stability are significantly offset by the combination of pore collapse and pore blockage that reduces the overall volumetric capacity of the processed MOF.^[^ ^16^ ^]^

Very recently, MOFs that are frequently termed “monolithic” but which can more accurately be described as “conformed” or “densified” have emerged as candidates that offer an appealing combination of simple preparation, practicality, and solutions to some or all the problems outlined above.^[^ ^4^, ^8^, ^17^ ^]^ This new class of MOF comprises densely packed primary nanocrystallites, agglomerated to form mm‐cm scale crystalline materials that omit the macroporosity of low‐density powder MOFs. Early reports have shown these to be highly stable mechanically and much easier to handle and recycle when compared to their powder counterparts.^[^ ^8^, ^17^ ^]^ Moreover, these materials, due to their macro nature, which is robust enough to tolerate the incorporation of active guests,^[^ ^17^, ^18^ ^]^ can easily be recovered from catalysis reaction media just using gravity filtration while their high densities render them contenders in gas storage.^[^ ^19^ ^]^ These benefits have combined to make them great options for industrial applications. The technique of growing monoliths without the need for external agents and high pressures was pioneered by Fairen‐Jimenez, who harnessed a general sol–gel approach, with the critical phase being slow drying of the intermediate gel at low temperature over a period of days rather than at elevated temperature over a period of hours.^[^ ^8^, ^17^, ^19^ ^]^ Early studies suggested that this slower drying step imparts epitaxial growth to the primary nanoparticles formed after the gelation, with the effect that robust, easily handled materials are formed. However, it has emerged that primary nanoparticle size is also a fundamental controller of both porosity^[^ ^19a^ ^]^ and monolithicity.^[^ ^17a^ ^]^ The sizes of these nanocrystallites can be varied by using reaction time, temperature, concentration, and modulators to manipulate reaction kinetics. They must, nevertheless, approximate to an optimal size, which is around 70 nm for the first reported _mono_MOF ZIF‐8 (though which can be as high as 150 nm in composite SnO_2_‐_mono_ZIF‐8),^[^ ^17b^ ^]^ but which can be as small as 10 nm for UiO‐66, which highlights the effects of creating the former at room temperature and the latter at 100 °C.^[^ ^8^, ^17^ ^]^ Crucially, powder formation results even with careful drying of the gel if primary nanocrystallites are not of the appropriate size.^[^ ^17^, ^20^ ^]^

Although more than 100 000 MOFs have been reported to date,^[^ ^4a^ ^]^ only around a dozen have been successfully synthesized as pristine monoliths.^[^ ^8^, ^21^ ^]^ These include ZIF‐8,^[^ ^17a^ ^]^ HKUST‐1,^[^ ^17c^ ^]^ UiO‐66,^[^ ^19a^ ^]^ *γ*‐CD‐MOF,^[^ ^22^ ^]^ Zr‐fumarate,^[^ ^23^ ^]^ MIL‐100(Fe),^[^ ^24^ ^]^ and MTV‐UiO‐66‐NH_2_.^[^ ^25^ ^]^ Meanwhile, it was recently shown that the same approach can be expanded to the field of covalent organic frameworks, with TPB‐DMTP‐COF reported.^[^ ^26^ ^]^ This slow development of the _mono_MOF field is majorly due to the lack of understanding of the variables that can affect monolithicity during the synthesis and drying steps. While some discussion of these factors has been attempted,^[^ ^27^ ^]^ it is only very recently that current thinking on the subject has been effectively collated.^[^ ^21^ ^]^ As noted above, most of the explanation for monolith formation has centered on post‐synthetic washing and drying procedures, with little discussion around the synthetic control of primary nanocrystallite formation and growth. Though it is recognized that increased electrostatic repulsion induces small primary nanocrystallites, extends gelation times,^[^ ^28^ ^]^ and so influences product porosity,^[^ ^29^ ^]^ these insights pre‐date the advent of _mono_MOFs. Meanwhile, some time has elapsed since the nucleation of ZIF‐8 nanocrystals was probed using time‐resolved static light scattering,^[^ ^30^ ^]^ in situ and ex situ X‐ray diffraction (XRD),^[^ ^31^ ^]^ transmission electron microscopy (TEM),^[^ ^31a^ ^]^ in situ small‐angle and wide‐angle X‐ray scattering (SAXS/WAXS)^[^ ^32^ ^]^ and X‐ray atomic pair distribution function (PDF) analysis.^[^ ^33^ ^]^ These works all pointed to the rapid growth of ZIF‐8 nanocrystals (over a few seconds to an hour). Accordingly, Polyzoidis et al. suggested overall ZIF‐8 yield be established within 15 s of mixing metal precursor and organic linker.^[^ ^31^, ^34^ ^]^ This being so, it was regarded as difficult to study particle nucleation, which led Terban et al. to impede the kinetics by modifying the solvent.^[^ ^33^ ^]^ However, even after replacing water with MeOH:H_2_O (1:1), nanocrystallites grew to 250 nm in just 30 min. Nevertheless, small and amorphous early‐stage ZIF‐8 particles could now be observed after 30 s as being ≈30–60 nm in size. Still, nucleation proved too rapid to shed further light on the rapidly formed and subsequently consumed ≈2 nm clusters observed by Cravillon et al. using in situ SAXS/WAXS. Created by monomer collision, these were proposed to underpin the transition from amorphousness to crystallinity during ZIF‐8 formation.^[^ ^32^ ^]^ Further insights have emerged recently, with Dok et al. establishing that small positively charged oligomeric prenucleation clusters bearing excess protonated linker form initially. A combination of harmonic light scattering and static NMR spectroscopy to probe the emergent solid‐state structure and mother liquor speciation, respectively, has established that these clusters aggregate by eliminating protonated linkers to give the neutral amorphous precursor particles.^[^ ^35^ ^]^

In the current report, because we are evolving an interest in the introduction of biomacromolecules to _mono_MOFs that means solvent compatibility becomes a concern, we manipulate solvent conditions to investigate the growth of primary nanocrystallites that go on to form a _mono_MOF. We focus on _mono_ZIF‐8, using aqueous‐based media instead of the previously reported approaches that have used purely organic solvents like ethanol.^[^ ^17a,b^ ^]^ New data significantly expand our scope for manipulating monolith growth and considerably extend the possibilities for the in situ encapsulation of entities by evolving monolith. Whereas guest incorporation has previously been shown for chemically inert metal oxide semiconductors, quantum dots, nanoparticles, and dyes (SnO_2_, CdSe, Au, sulforhodamine 640),^[^ ^17^, ^18^, ^36^ ^]^ we now establish conditions that promise the doping of perovskites, biomolecules, and solvent‐sensitive organic molecules. Enzymes are typically denatured when exposed to unsuitable pH or solvents like ethanol,^[^ ^37^ ^]^ while many perovskites are also quenched or prone to agglomerate in the presence of such a polar solvent.^[^ ^38^ ^]^ In the case of organics, stability may be less of an issue, though solubility or miscibility in a specific solvent can remain a challenge. To expand the portfolio of candidates for in situ encapsulation into _mono_MOF via a de‐novo approach, we, therefore, seek to understand the effect of solvent choice on nanocrystallite growth kinetics and monolith formation in ZIF‐8. To this end, achieving an ultra‐low rate of reaction promotes the study of nucleation and particle growth. Our monolith exhibits mesoporosity, which also offers the possibility of post‐synthetic doping by guest species.

## 2 Results and Discussion

**Figure 1:**
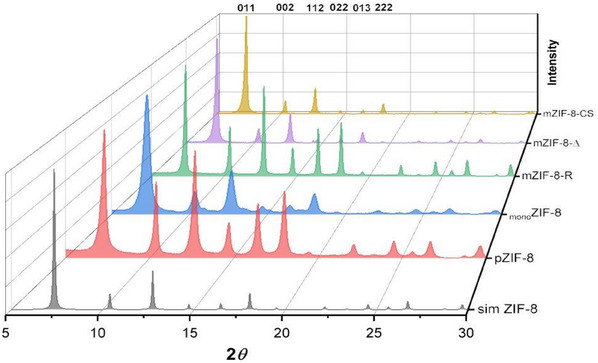
Selected PXRD data, including hkl reflections, for samples in Experimental Section Table 4 (see below). — Simulated ZIF‐8; — _mono_ZIF‐8 (entry 1); — pZIF‐8 (entry 3); — mZIF‐8‐R (entry 11); — mZIF‐8‐Δ (entry 12); — mZIF‐8‐CS (entry 13).

The synthesis of _mono_ZIF‐8 was first reported by Tian et al. in 2015.^[^ ^17a^ ^]^ By directly introducing preformed semiconducting nanoparticles into that synthesis it was next possible to demonstrate the in situ encapsulation of guests into a monolithic host.^[^ ^17b^ ^]^ Recently, interest in making the synthesis of _mono_ZIF‐8 compatible with the inclusion of biomolecular guests such as enzymes, which can be incompatible with high levels of ethanol, has led to attempts to change the reaction medium to water whilst keeping other parameters the same as for previous work in our group.^[^ ^39^ ^]^ Accordingly, the 1:8 combination of Zn(NO_3_)_2_·6H_2_O and 2‐mIm in ethanol (see Experimental Section below for synthetic details, Table 4 entry 1) was modified to use water instead, but yielded an impure product that incorporated crystalline ZIF‐8 and at least one other unidentified crystalline by‐product (Table 4 Entry 2 and Figure S2, Supporting Information). This approach having failed, the Zn:2‐mIm ratio was increased from 1:8 to 1:40 (Table 4 entries 3–13), following the work of Zhu et al.^[^ ^40^ ^]^ In each case, the sol–gel process followed by slow drying of the initially formed gel gave a single isolable crystalline product that was analyzed as pure ZIF‐8 by PXRD (see **Figures** **1** and S2, Supporting Information). In all data, peaks at 7.4° (011), 10.5° (002), 12.8° (112), 14.7° (022), 16.5° (013) and 18.1° (222) match with the simulated diffraction pattern.^[^ ^41^ ^]^

While entry 3 represented only the replacement of ethanol by water as both reaction and washing media, entries 4–6 attempted primary nanocrystallite size control by aging the sol for 3, 24, or 48 h, respectively,^[^ ^42^ ^]^ following the report by Taddei et al. that a primary particle size reduction can be induced by aging the reaction mixture. Entries 7–8 explored the use of ethanol as a post‐synthetic washing medium presuming that solvent‐sensitive biomolecules would be protected from the environment through their prior encapsulation by the MOF primary particles. This being so, solvent exchange should not affect the activity of the guests. Entries 7–8 probed differences in product formation when gels were washed in gradually increasing proportions of EtOH or in absolute EtOH. Entries 9–11 employed mixed organic‐aqueous reaction and washing media, exploring the use of DMF, DMSO, and EtOH at room temperature. Many organic solvents show denaturing effects toward proteins. However, at lower concentrations, the three solvents tested can do minimal damage to proteins and in some cases can even augment catalytic activity.^[^ ^43^ ^]^ Moreover, they offer the ability to manipulate electrostatic interactions between particles in the reaction medium and, through this, proximity and reaction rate.^[^ ^28^ ^]^ In entries 9–11, colloid formation proceeded smoothly, yet it was noticeable that *gel* formation was significantly slower when using EtOH, necessitating an increase in reaction time from 0.5 to 120 h at ambient temperature. Attempts to limit this extension of reaction duration followed, with two approaches investigated based on the known effects of temperature and ionic salts on colloid aggregation according to Derjaguin–Landau–Verwey–Overbeek (DLVO) theory.^[^ ^44^ ^]^ First, maintaining the parameters from entry 11, an elevated temperature reaction (entry 12) yielded a gel after 96 h. Second, CaSO_4_, a recognized flocculant capable of disturbing the diffused double layer around the colloid particles, thinning their solvation sphere, and promoting their aggregation,^[^ ^45^ ^]^ was introduced to the 15% v/v EtOH–H_2_O solution of Zn(NO_3_)_2_ before mixing with 2‐mIm to give a gel after 72 h (entry 13). Lastly, given the ability of PXRD to identify only crystallographically ordered phases, combustion microanalysis, and ICP‐OES were used to verify compositions. Monoliths prepared using 15% v/v EtOH−H_2_O generally showed excellent agreement with the theory (Table S1, Supporting Information). In the case of mZIF‐8‐CS, marginally less good agreement corresponds to the observation (see below) that a mixture of two (eventually separately solid) products is obtained. Interrogation of the major of the two products by ICP‐OES and PXRD suggests that a small amount (3.5 wt.%) of Ca is incorporated with mainly monolithic ZIF‐8 (see Figure S1 and Table S1, Supporting Information). In contrast, compositional analysis suggests the minor product to be Ca‐rich with minor contamination by unreacted zinc and imidazole starting materials (see below).

Consideration of the outcomes of reactions in entries 3–13 enabled several conclusions to be drawn about the nature of pure products formed using 1:40 Zn:2‐mIm. The opacity of pelletized samples in entries 3–6 suggested the poor densification of primary nanocrystallites irrespective of aging when the solvent used for reaction and washing was exclusively water. Prior reports have established that slow evaporation of reaction solvent in the presence of unreacted starting material promotes more efficient primary nanocrystallite interaction by effectively allowing the reaction to continue.^[^ ^19a^ ^]^ However, current data point to ineffective epitaxial growth of primary nanocrystallites in water, with the maintenance of optically visible barriers between the nanocrystallites giving a highly opaque pellet. This suggests inappropriately sized primary nanocrystallites that resist effective densification. This conclusion is reinforced by the pellets having the matt appearance typical of compacted powders rather than the luster normally associated with monolithicity.^[^ ^17^, ^19^ ^]^ In line with this, where water is the exclusive solvent, pellets invariably lack the qualitative robustness of monoliths. Entries 3–8 are therefore considered to report powders (pZIF‐8‐x).

Moving to mixed organic‐aqueous reaction media gave contrasting results (entries 9–11). The use of 15% v/v Sol−H_2_O (Sol = DMF, DMSO) yielded only matt pellets. In contrast, for Sol = EtOH, a lustrous pellet was obtained. That it was translucent rather than transparent may have several origins, including differing primary nanocrystallite size and packing efficiency,^[^ ^17b^ ^]^ crystal morphology,^[^ ^46^ ^]^ and organic contamination.^[^ ^47^ ^]^ In the current case, the phenomenon likely reflects the incorporation of unreacted 2‐mIm (*viz*. the high Zn:2‐mIm ratio used in the synthesis and the observation that washing with MeOH to expel organics causes the lustrous pellet obtained using EtOH to become more opaque). Qualitative assessment of the robustness of these three samples is consistent with DMF and DMSO giving powders, but EtOH successfully yielding _mono_ZIF‐8 under ostensibly aqueous conditions. Lastly, having established that the use of 15% v/v EtOH−H_2_O gives promising results, the unexpected extension of gel formation time from 0.5 to 120 hours was investigated (entries 12–13). In the first reaction, the temperature was elevated to 50 °C, and in the latter, CaSO_4_ was introduced (0.5 eq. w.r.t. Zn). Gels were obtained after 96 and 72 h reactions, respectively, and both dried to give monolithic ZIF‐8. In the last case, visual inspection suggests a separate phase (clearly visible as a white residue in Figure S1E, Supporting Information). ATR FTIR and PXRD clarify that this is not ZIF‐8 (Figure S2, Supporting Information). Instead, a comparison with precursors (2‐mIm, Zn(NO_3_)_2_ and CaSO_4_·2H_2_O) suggests that it is a mixture of unreacted nitrate and calcium sulfate (Figures S3 and S4, Supporting Information).

**Figure 2:**
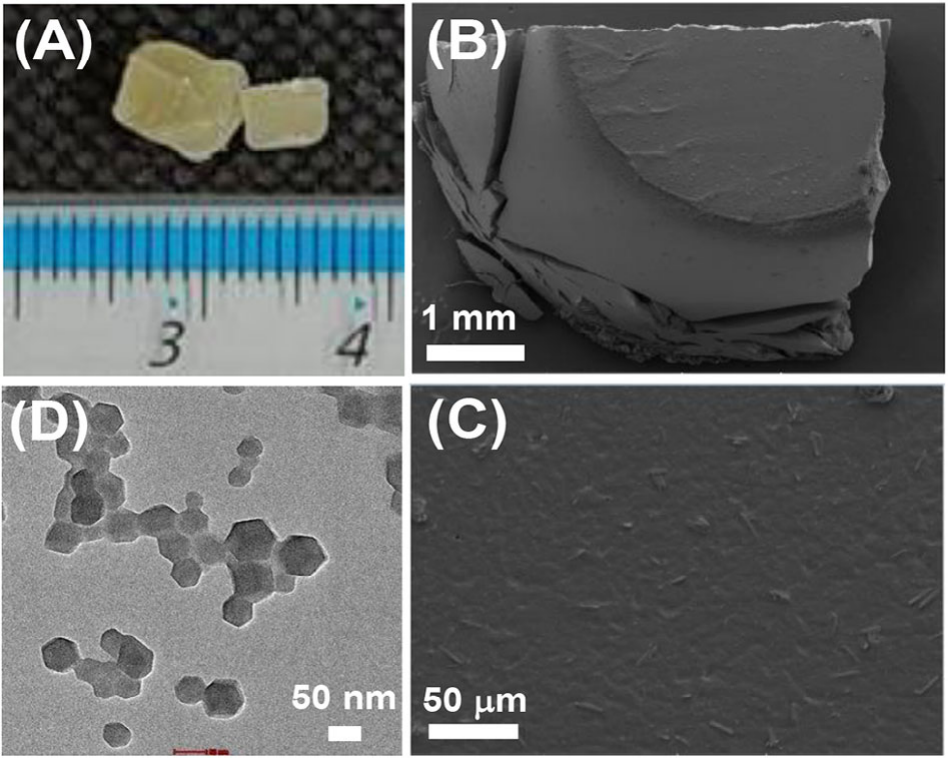
Representative analysis of mZIF‐8‐Δ. A) Optical image. B,C) low and high magnification SEM images, and D) TEM imaging of a diluted reaction aliquot.

SEM imaging corroborates the optical and qualitative mechanical assessments described above. At high magnification, it is evident that the monolith (**Figure** **2**B) is not a loose agglomeration of nanoparticles, but a single entity with a flat surface (Figure 2C) similar to that previously reported,^[^ ^17a^ ^]^ and matching well with reference _mono_ZIF‐8 prepared here (Table 4, entry 1 and Figure S5A,B, Supporting Information). In contrast, pZIF‐8 (Table 4, entry 3) produced distinctly contrasting high‐magnification SEM data (Figure S5C,D, Supporting Information). TEM analysis helps rationalize the SEM observations (Figure S11, Supporting Information). Hence, samples pZIF‐8 and pZIF‐8‐b‐d are all attributed powder morphologies based on pellet appearance and qualitative mechanical properties. In line with this, the primary nanocrystallites imaged by aliquoting a small amount of diluted gel immediately post‐reaction onto a Cu microscope grid show very large particles, with the aging of the sol demonstrating just a limited ability to decrease their size (from ≈670 to ≈590 nm). These data explain why not only pZIF‐8 but also samples incorporating aged sols (pZIF‐8‐b‐d) fail to form monoliths. Unlike in previous reports,^[^ ^42^ ^]^ in this work, the aging of the combined sol had no statistically detectable effect on primary particle size. In contrast, the use of 15% v/v EtOH−H_2_O for extended reaction times plainly gives primary nanocrystallites of a size previously documented to yield monoliths.^[^ ^17a,b^ ^]^ Representatively, Figure S11E (Supporting Information) shows a significant decrease to 55±10 nm of the mean size of primary particles obtained after 96 h at 50 °C during the preparation of mZIF‐8‐Δ. This explains the ability of the 15% v/v EtOH−H_2_O samples to display monolith formation (Figure 2B). Attempts to probe the formation and evolution with the time of primary nanocrystallites in reaction mixtures that will go on to form mZIF‐8‐R and mZIF‐8‐∆ reveal interesting behavior. While images substantiate the mean particle sizes reported in Figure S11 (Supporting Information), they also demonstrate the rapidity with which primary nanocrystallites form irrespective of the time required for gelation. Hence, for mZIF‐8‐R primary particles are essentially formed after just 15 min., albeit their shapes lack the definition that emerges after 72 h (Figure S12, Supporting Information). Data is clearer for mZIF‐8‐∆, where poorly defined primary particles are seen after 1 h. While these remain about the same size as particles imaged in aliquots from the reaction after it has proceeded over 96 h (see below), they plainly evolve, developing the hexagonal shapes characteristic of Figure S11E (Supporting Information) after 24 h (Figure S13, Supporting Information).

Having posited monolithicity based on qualitatively assessing the mechanical strength of samples prepared in 15% v/v EtOH−H_2_O, we sought to quantify these properties to investigate the ability of samples produced here to withstand the stresses of friction, compression, or vibration synonymous with applications. Nanoindentation established that the magnitudes of the indentation modulus, E*, and Hardness, *H*, of mZIF‐8‐∆ (E* = 3.8 ± 0.1 GPa, *H* = 0.56 ± 0.03 GPa; Figures S20–S22, Supporting Information) compared closely with those of single crystalline ZIF‐8^[^ ^48^ ^]^ but were greater than those of previously published _mono_ZIF‐8 (E* = 3.6 ± 0.2 GPa, *H* = 0.43 ± 0.03 GPa)^[^ ^17a^ ^]^ and SnO_2_@_mono_ZIF‐8 (3.30 ± 0.01 and 0.44 ± 0.01 GPa).^[^ ^17b^ ^]^ In line with these latter values, a recent report proposed an initial grain boundary sliding mechanism followed by localized partial framework failure to explain mechanical deformation in a sample of monolithic ZIF‐8 with *H* = 0.45 ± 0.02 GPa.^[^ ^49^ ^]^ The structurally stiffer characteristics of current mZIF‐8‐∆ and its greater resistance to permanent deformation are likely attributable to the much‐reduced rate of gelation allowing the primary particles to pack more efficiently.^[^ ^50^ ^]^ The thermal stabilities of mZIF‐8 samples prepared with 15% v/v EtOH−H_2_O were measured by TGA (Figure S23, Supporting Information). At ≈600 °C decomposition temperatures for each proved consistent with previously reported monolithic ZIF‐8.^[^ ^17a,b^ ^]^ Small weight losses of 14% and 20% for mZIF‐8‐∆ and mZIF‐8‐CS respectively, at up to ≈250 °C can be attributed to ethanol and water trapped in the materials^[^ ^17a^ ^]^ though the magnitude of the drop is greater than in the prior art and trapped 2‐mIm (now used in higher proportions) is not ruled out.^[^ ^51^ ^]^ Importantly, the thermal stability of none of the monoliths prepared here was altered by the significantly different rates with which primary particles agglomerated in mixed‐solvent systems.

**Figure 3:**
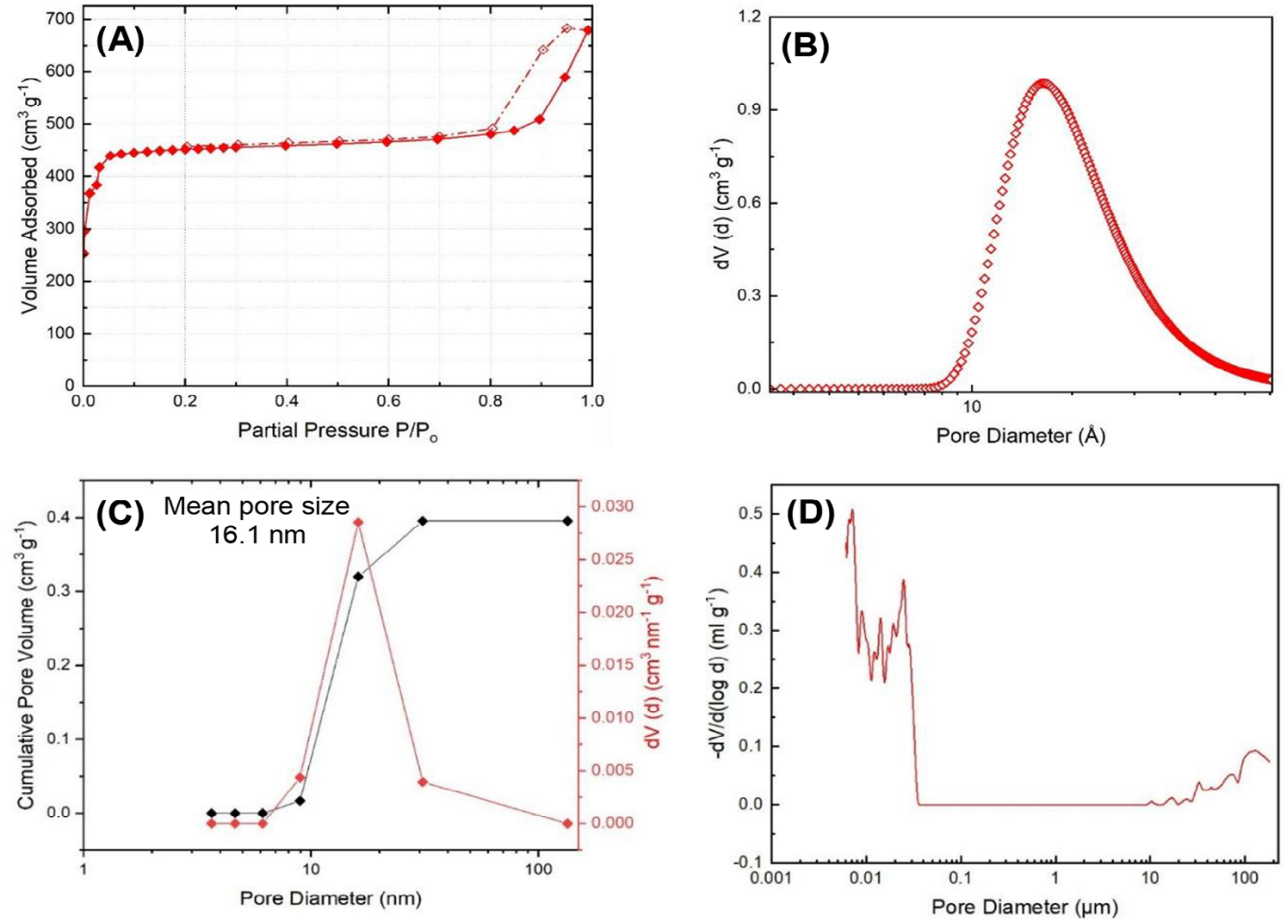
For mZIF‐8‐Δ: A) N_2_ adsorption‐desorption isotherm collected in the range 0–1 bar at 77 K (◆ adsorption, 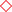 desorption), B) Corresponding microporous DA PSD, C) BJH mean (◆) and PSD (◆) obtained from the N_2_ isotherm data, and D) Macroporous PSD obtained from Hg Porosimetery.

N_2_ adsorption‐desorption isotherms, BET surface areas, pore size distributions (PSDs), total pore dimensions, and micropore volumes are compared in **Figures** **3** and S24 (Supporting Information) and **Table** **1**. As expected, analysis shows a Type‐I isotherm for pZIF‐8, which is characteristic of microporous materials. On the other hand, mZIF‐8‐R and mZIF‐8‐∆ each exhibit a hysteresis loop at higher pressure, both revealing a Type‐IV isotherm consistent with the presence of mesoporosity. The lower S_BET_ seen for mZIF‐8‐R is suggested to result from the slower nucleation expected and higher polydispersity seen in comparison to mZIF‐8‐∆. This last shows an essentially identical value of S_BET_ to the original ethanolic monolith reported by Tian et al. This is despite the fact that, while that ethanolic monolith was microporous, the current sample of _mono_ZIF‐8 is mesoporous with a BJH mean pore size of 16 nm. Behaving differently from these monoliths, mZIF‐8‐CS mimics the adsorptive and desorptive properties of powder ZIF‐8, which may be due to the presence of trapped CaSO_4_ in the mesopores of the MOF. Not only do its Type‐I isotherm and identical total pore and micropore volumes point to microporous ZIF‐8,^[^ ^17^, ^52^ ^]^ but entrapment of residual flocculant would explain its lower S_BET_ powder morphology. This is demonstrated by pZIF‐8 in Figure S27A (Supporting Information) and contrasts with mZIF‐8‐R. The latter is ≈3 times denser on account of its monolithicity, with its density of 0.90 g cm^−3^ compared with that of ≈1.1 g cm^−3^ previously reported for microporous, ethanolic monolith^[^ ^17a^ ^]^ and suggesting the inclusion of just a small fraction of mesopores currently (Figure S27B, Supporting Information). Clearly, then, monolithic ZIF‐8 is achievable despite the present use of water as the main solvent. As noted above, elevated temperature induces a remarkable S_BET_ and higher proportion of mesopores in mZIF‐8‐∆ (Figure S27C, Supporting Information). This last observation is in line with the lower density (roughly twice that of powder ZIF‐8) and is consistent with previous reports on the relationship between density and mesoporosity in monolithic UiO‐66.^[^ ^19a^ ^]^ Lastly, the inclusion of CaSO_4_ has been argued to promote pore blockage (see above). Correspondingly, and in contrast to mZIF‐8‐R and ‐∆, mZIF‐8‐CS lacks accessible mesopores (Figure S27D, Supporting Information). Whilst this is reflected in *W*_Tot_/*W*_0_, ρ_b_ is close to that seen in mZIF‐8‐∆ on account of monolithicity.

**Table 1:** Gravimetric BET area (S_BET_), total pore diameter (D_Tot_), total volume (W_Tot_), micropore volume (W_0_), bulk density (ρ_b_), and volumetric BET area (S_BET_ (vol)) for ZIF‐8 samples prepared in this work.

	S_BET_ [m^2^ g^−1^]	D_Tot_ [nm]	W_Tot_ [cm^3^ g^−1^]	W_0_ [cm^3^ g^−1^]	ρ_b_ [g cm^−3^]	S_BET_ [vol] [m^2^ cm^−3^]
pZIF‐8	1450	1.3^b)^	0.55	0.53	0.35^a)^	508
mZIF‐8‐R	1027	16.1^c)^	0.61	0.50	0.90	924
mZIF‐8‐Δ	1421	16.1^c)^	1.05	0.71	0.76	1080
mZIF‐8‐CS	1124	1.5^b)^	0.66	0.66	0.71	798

^a)^ As reported;^[^ ^17a^ ^]^

^b)^ DA mean pore size;

^c)^ BJH mean pore size

As noted above, changing the solvent system from pure ethanol or pure water to a 15% v/v EtOH–H_2_O solvent mixture induces the rate at which primary particles nucleate to be much reduced. In the ethanolic (_mono_ZIF‐8) and aqueous (pZIF‐8) syntheses, reaction times of 30 min. were sufficient to form a sol which when centrifuged (5500 rpm for 12 min.) separated into gel and clear supernatant. When the same reaction was tried with 15% v/v EtOH–H_2_O, the transformation proceeded much more slowly. It was possible to visually observe colloid formation within minutes of mixing and TEM analysis (see above) pointed to the rapid formation of primary particles. Analysis of these after short reaction times (Figures S12 and S13, Supporting Information) suggests particle sizes superficially similar to those exhibited at the end of the reaction. That is, while the particles significantly improve their definition with time, they do not appear to grow significantly throughout the reaction. This ex situ analysis should be treated with caution, however, on account of effects that appear to result from the drying of the sample on the TEM grid.^[^ ^53^ ^]^ These are most evident at low reaction times, where the slow evaporation of water combined with the presence of unreacted organic feedstock (visible in Figure S13 in Supporting Information at up to 24 h reaction time but absent thereafter) is credited with allowing reaction and particle growth to continue on the grid. In line with this, the inability after 30 min. to isolate gel from the mZIF‐8‐R system, even using centrifugation parameters of 9000 rpm for 120 min. Strongly suggests an absence of large particles in situ. Instead, extending the reaction to 120 h enables successful centrifugal gel isolation, albeit the supernatant is still cloudy (see below). To purify the supernatant whilst hopefully reducing reaction time, the reaction was repeated at 50 °C (mZIF‐8‐Δ). Indeed, this has made it possible to isolate the gel after 96 h, with ex situ TEM investigation of primary nanocrystallites once again revealing drying effects in initial aliquots and well‐formed, hexagonal particles 55 ± 10 nm in size at the point of gelation (Figures S11 and S13, Supporting Information). The view of primary nanocrystallite behavior developed for both mZIF‐8‐R and mZIF‐8‐Δ is consistent with prior art that saw the initial formation of particles ≈2 nm in size proposed.^[^ ^32^ ^]^ Additionally, in the case of mZIF‐8‐Δ, a clear supernatant can be observed, which also suggests more homogeneity in the size of the colloidal particles (see below). Lastly, to prepare mZIF‐8‐CS, ionic salt CaSO_4_ was introduced to expedite sol formation through its documented ability to function as a flocculant.^[^ ^45^ ^]^ This further improved the reaction rate, with gel separated from clean supernatant after only 72 h.

**Figure 4:**
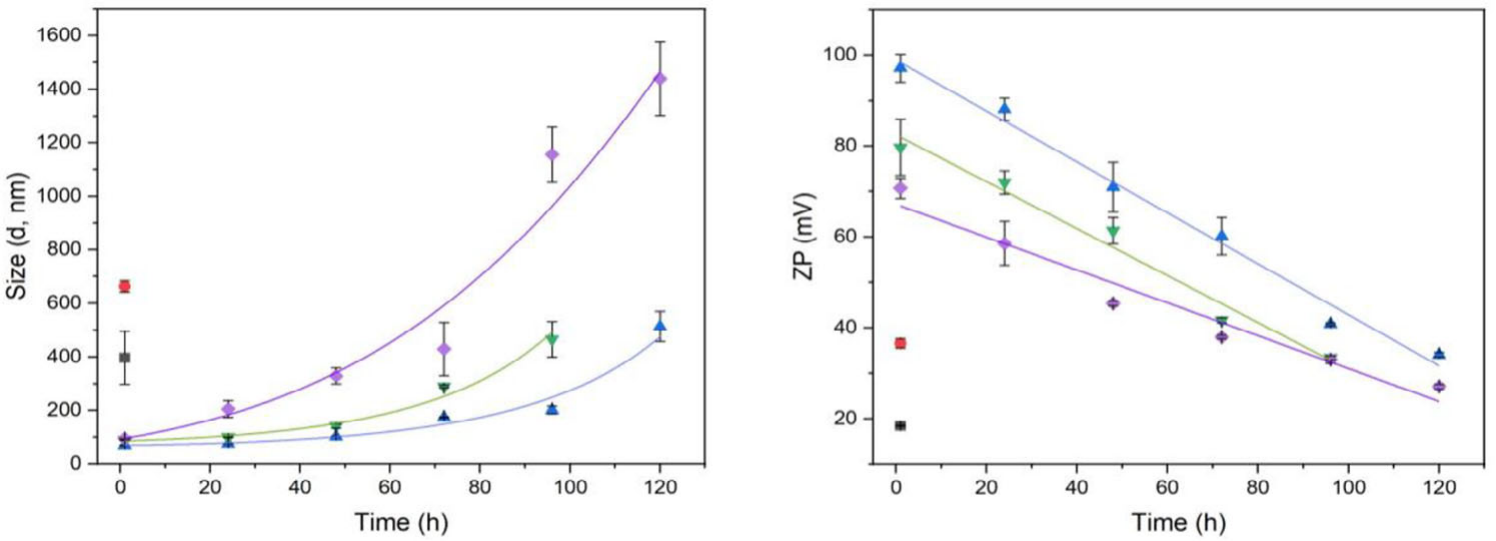
Graphical representations of (left) hydrodynamic particle size (nm) and (right) ζ‐potential (mV) against time (h) for aliquots from a range of reactions at 1‐120 h. All readings are triplicated. ■ EtOH. ● H_2_O. ▲ EtOH−H_2_O, room temperature. ▼EtOH−H_2_O, 50 °C. ◆ EtOH−H_2_O, CaSO_4_.

The ambiguity over primary nanocrystallite formation caused by drying effects when using ex situ analysis meant investigation of the kinetics of MOF primary particle nucleation was undertaken in situ using DLS to derive particle hydrodynamic radius from diffusion coefficient through the Stokes‐Einstein relationship.^[^ ^54^ ^]^ Aliquots were taken from a range of reaction mixtures at times of 1–120 h. Focusing on reactions in 15% v/v EtOH–H_2_O, samples revealed exponential growth in hydrodynamic diameters for each of mZIF‐8‐R, mZIF‐8‐Δ, and mZIF‐8‐CS, with the last showing the fastest rate of nucleation of the three (**Table** **2** and **Figure** **4**). In each system, data correlate both with the prior literature and with TEM analysis. Representatively, mZIF‐8‐∆ reveals an initial hydrodynamic radius of ≈90 nm, of which the majority can be attributed to the surface double layer.^[^ ^55^ ^]^ Data suggest particle growth to ≈465 nm at the point of gelation, which is consistent with primary nanocrystallites ≈60 nm in size encapsulated by a still substantial double layer. Lastly, in all three cases, cumulant particle size data, *Z*_avg_, generally agree with the major peak (Table S2 and Figure S28, Supporting Information). The only exception to major peak and *Z*_avg_ correlation is in the case of mZIF‐8‐R where, at 120 h, *Z*_avg_ approximates to half of the major peak. This suggests polydispersity and the presence of smaller particles. Such a view is reinforced by the observation that centrifugation only partially separated the gel, leaving a cloudy supernatant (Figure S29, Supporting Information). The increase in the hydrodynamic size seen for primary particles in mixed‐solvent systems corresponds to the linear decrease in ζ‐potential seen in each reaction mixture with time (**Table** **3** and Figure 4). When ζ‐potential decreases to below ≈40 mV, it becomes straightforward to separate the colloid under centrifugal force (Table S3, Supporting Information), and this trend is reflected in the reaction times deployed for mixed‐solvent systems in this work.

**Table 2:** Hydrodynamic diameter distribution (nm) for aliquots from a range of reactions at 1–120 h.

Hours	Peak [d, nm]				
	_mono_ZIF‐8	pZIF‐8	mZIF‐8‐R	mZIF‐8‐Δ	mZIF‐8‐CS
1	396.4	663.5	70.5	89.3	95.4
24			75.6	100.2	205.6
48			102.1	141.4	328.9
72			174.3	289.6	429.8
96			201.4	465.4	1156.1
120			513.4		1440

**Table 3:** ζ‐Potential (mV) for aliquots from a range of reactions at 1–120 h.

Hours	ZP [mV]				
	_mono_ZIF‐8	pZIF‐8	mZIF‐8‐R	mZIF‐8‐Δ	mZIF‐8‐CS
1	18.4	36.6	97.2	79.7	70.7
24			88.1	72	58.6
48			70.9	61.4	45.4
72			60.2	41.7	37.9
96			40.8	33.4	33.0
120			34.1		27.1

The reduced rate at which isolable gel flocculates in this work can be understood in terms of very recent work by Dok et al. that shed light on the transition from very small initially created prenucleation clusters to amorphous intermediates in the early stages of reaction.^[^ ^35^ ^]^ In this context, Venna et al. have used in situ PXRD and TEM data to suggest medium‐range ordered species.^[^ ^31a^ ^]^ This view was reinforced by the results of SAXS and XPDF analysis,^[^ ^32^, ^33^ ^]^ which led to the proposition of an early‐stage particle size of ≈2 nm when crystallinity was emerging in the evolving system. These data, all obtained for the formation of powdered MOFs, are nevertheless in consensus with our experiments on the formation of a monolithic morphology, where we observe a lack of deposit after the initial colloid formation step. Notably, it was previously reported that ZIF‐8 formation in a mixed solvent system (1:1 MeOH:H_2_O) afforded a reduced reaction rate,^[^ ^33^ ^]^ albeit not so slow as in the current study. Based on these and our new data, we consider that the stability of intermediates in the solvent mixture used presently is the main reason the overall nucleation rate is impeded.

## 3 Conclusion

Work reported here has established for the first time that control over the monolithicity with which a MOF grows can be extended beyond post‐synthetic washing and drying procedures. Instead, the combination of Zn^2+^ with 2‐mIm in solvent mixtures allows the colloid formation to proceed smoothly, yet it delays gel formation. Using a 15% v/v EtOH−H_2_O solvent mixture necessitates extending the reaction time for up to 120 h. In the case of mZIF‐8‐∆, the product matched benchmark S_BET_ results for microporous monolith despite the introduction of mesopores suitable for applications requiring guest inclusion. Moreover, it proved more resistant to permanent deformation than previously reported ZIF‐8 monoliths. This was attributed to the highly efficient packing of uniform primary particles ≈60 nm in size; a view substantiated by in situ analysis techniques. These have allowed the correlation of hydrodynamic nanocrystallite size with ζ‐potential, pointing to the stability of intermediates in the EtOH−H_2_O system controlling the overall nucleation rate. We hypothesize that nucleation is caused by changes to solvent mixture composition as EtOH preferentially evaporates (to be expected, but see also Figure S31, Supporting Information) and are initiating a computational study into this. At the same time, having fabricated mesoporous mZIF‐8 using biocompatible media at either ambient temperature or 50 °C, preliminary efforts at incorporating formate dehydrogenase, glutamate dehydrogenase, and nicotinamide adenine dinucleotide (NAD) are being developed. Preliminary UV–Vis absorbance data for NADH in the presence of mesoporous mZIF‐8‐∆ point to a 4‐fold reduction in absorbance at λ_max_ for the enzyme co‐factor over 6 h (Figure S32, Supporting Information). This prima facie suggests successful monolith doping with NADH. The conversion of CO_2_ and glutamic acid to industrial feedstock formic acid in its own right or as the initial step of the cascade synthesis of methanol^[^ ^40^ ^]^ will then be studied using a monolithically immobilized catalyst array. Both C_1_ products represent value‐added chemicals whose synthesis from CO_2_ would highlight the potential benefits of efficient carbon capture and utilization.

## 4 Experimental Section

### Materials

Zinc nitrate hexahydrate (98%) was purchased from Acros Organics. 2‐methylimidazole (99%) and calcium sulfate dihydrate were from Sigma‐Aldrich, and ethanol (absolute) was obtained from Fisher Scientific. All chemicals were used as received. Deionized water was used throughout.

### Syntheses of ZIF‐8

Samples prepared as described in a‐h below. Any samples requiring activation were soaked in MeOH overnight, then isolated using centrifugation and degassed overnight at 110 °C in a vacuum oven.

- _mono_ZIF‐8 was synthesized based on a modified preparation.^[^ ^39^ ^]^ Zinc nitrate hexahydrate Zn(NO_3_)_2_·6H_2_O (0.293 g, 0.985 mmol) was dissolved in ethanol (20 mL). 2‐mIm (0.649 g, 7.90 mmol) was dissolved separately in ethanol (20 mL). The solutions were combined and then stirred for 15 min. The resulting sol was centrifuged (5500 rpm, 10 min.). The collected gel was washed in ethanol (20 mL, 3 times) under ultrasonication (5 min.) and the purified gel left to dry slowly at ambient temperature. (See Table 4 Entry 1);
- pZIF‐8‐a was prepared as per synthesis (a) notwithstanding that ethanol was replaced by water throughout. (See Table 4 Entry 2);
- pZIF‐8 was synthesized based on a modified literature preparation.^[^ ^40^ ^]^ An aqueous solution of Zn(NO_3_)_2_·6H_2_O (0.185 g, 0.62 mmol, 2 mL) was mixed with one of 2‐methylimidazole (2‐mIm, 2.05 g, 25.0 mmol, 20 mL) under stirring at ambient temperature. The mixture instantly turned milky. It was stirred for 30 min. to give a sol that was isolated as a gel by centrifuging at 5500 rpm for 12 min. Then washed with deionized water (20 mL, 3 times) under ultrasonication (5 min.). The gel was left at ambient temperature until completely dry, per Table 4 Entry 3;
- pZIF‐8‐b/c/d were prepared as per synthesis (c) notwithstanding that after 30 min. of stirring the sol was aged for 3, 24, or 48 h before centrifugation. (See Table 4 Entry 4–6);
- pZIF‐8‐e/f were prepared as per synthesis (c) notwithstanding washing steps were modified to ethanol (20 mL, 3 times) for sample (e) and 15% v/v EtOH‐H_2_O, 30% v/v EtOH‐H_2_O and then 50% v/v EtOH‐H_2_O for (f). (See Table 4 Entry 7–8);
- pZIF‐8‐g/h were prepared by modifying preparation (c) to use solutions of Zn(NO_3_)_2_·6H_2_O and 2‐mIm in 15% v/v DMF or DMSO in water. In each case, the colloid was centrifuged at 5500 rpm for 20 min. and washing was with 15% v/v DMF or DMSO in water under ultrasonication. (See Table 4 Entry 9–10);
- mZIF‐8‐R and mZIF‐8‐Δ were prepared as per synthesis (f) using 15% v/v EtOH in water. For the former system, the reaction was by stirring for 120 h at ambient temperature. For the latter system, 96 h at 50 °C was used instead. (See Table 4 Entry 11–12);
- mZIF‐8‐CS was prepared as mZIF‐8‐R notwithstanding that anhydrous CaSO_4_ (0.05 g, 0.37 mmol) was added to the solution of Zn(NO_3_)_2_ at the outset. The reaction mixture was stirred at ambient temperature for 72 h. (See Table 4 Entry 13).

**Table 4:** Samples of ZIF‐8 synthesized in this work (p = powder, m = monolith, RT = room temperature, Δ = heat, CS = calcium sulfate). ^a)^Molar ratio.

Entry	Sample	Solvent	Zn:2‐mIm^a)^	Conditions	Aging	Washing	Result
1	_mono_ZIF‐8	EtOH	1:8	0.5 h	0 h	20 mL EtOH × 3	Monolith
2	pZIF‐8‐a	H_2_O	1:8	0.5 h	0 h	20 mL H_2_O × 3	Mixture
3	pZIF‐8	H_2_O	1:40	0.5 h	0 h	20 mL H_2_O × 3	Powder
4	pZIF‐8‐b	H_2_O	1:40	0.5 h	3 h	20 mL H_2_O × 3	Powder
5	pZIF‐8‐c	H_2_O	1:40	0.5 h	24 h	20 mL H_2_O × 3	Powder
6	pZIF‐8‐d	H_2_O	1:40	0.5 h	48 h	20 mL H_2_O × 3	Powder
7	pZIF‐8‐e	H_2_O	1:40	0.5 h	0 h	15% EtOH→ 30% EtOH →50% EtOH (in H_2_O, 20 mL)	Powder
8	pZIF‐8‐f	H_2_O	1:40	0.5 h	0 h	20 mL EtOH × 3	Powder
9	pZIF‐8‐g	15% DMF in H_2_O	1:40	0.5 h	0 h	20 mL 15% DMF in H_2_O × 3	Powder
10	pZIF‐8‐h	15% DMSO in H_2_O	1:40	0.5 h	0 h	20 mL 15% DMSO in H_2_O × 3	Powder
11	mZIF‐8‐R	15% EtOH in H_2_O	1:40	120 h	0 h	20 mL 15% EtOH in H_2_O × 3	Monolith
12	mZIF‐8‐Δ	15% EtOH in H_2_O	1:40	96 h (Δ)	0 h	20 mL 15% EtOH in H_2_O × 3	Monolith
13	mZIF‐8‐CS	15% EtOH in H_2_O	1:40	72 h (with CaSO_4_)	0 h	20 mL 15% EtOH in H_2_O × 3	Monolith

### Powder XRD

All Powder XRD data were recorded on a Panalytical Empyrean diffractometer fitted with an X'celerator detector and using a Cu‐Kα_1_ (λ = 1.540598 Å) source operating at 40 kV and 40 mA with a step size of 0.02°. Monoliths were crushed into a fine powder for powder diffraction analysis.

### Elemental Analysis

C, H, N analysis was performed on an Exeter Analytical Inc. CE‐440 Elemental Analyzer, with a combustion temperature of 975 °C. Inductively coupled plasma‐optical emission spectroscopy (ICP‐OES) was used for Zn quantification and was performed on a Thermo Fisher Scientific iCAP7400 Duo ICP‐OES spectrometer using Qtegra software. ICP standards and tetramethyl ammonium hydroxide were purchased from Sigma–Aldrich. Nitric acid (Trace metal grade) was from Fisher Scientific, and water (Trace metal grade) was from Honeywell/Riedel‐de Haen. Activated MOFs (2 mg) were digested in nitric acid (5 mL), and diluted with water (5 mL). A 0.5 mL aliquot was then diluted to 10 mL with water. Standard curves in the range of 0.01–10 ppm were prepared by diluting commercial standard with 2% nitric acid solution.

### FTIR Spectroscopy

FTIR spectra were acquired using a Thermo Scientific Nicolet iS50 spectrometer operating in attenuated total reflection (ATR) mode.

### Electron Microscopy

Low magnification scanning electron microscope (SEM) images were captured using TESCAN MIRA3 FEG‐SEM and the images were processed using the Oxford Instruments AzTec Suite. The samples were placed on an Al‐stub and sputter‐coated with a 20 nm layer of chromium before placing into the instrument. High magnification SEM images, elemental data, and EDS analysis were done on JEOL IT500HR/LA high‐resolution SEM operating at 2 kV. TEM images were collected using Talos F200X G2 and data were analyzed using FEI TEM Imaging and Analysis and EDS data were processed using Velox. Lower magnification images were captured using a JEOL JEM‐2010 microscope operating at 200 kV with a space resolution of 0.24 nm. Primary nanocrystallite size distributions were calculated using ImageJ. 1 mL of sol was collected and washed with 20 mL fresh solvent (H_2_O for pZIF‐8 and 15% v/v EtOH‐H_2_O for mZIF‐8‐X) and centrifuged (5500 rpm for 10 min.). The supernatant was disposed of, and the remaining gel was dispersed in a fresh solvent (4 mL). In each case, 4 µL of the suspension was transferred to a continuous carbon‐coated Cu grid and the solvent was allowed to evaporate before the sample was mounted in the instrument. Microscopic analysis of dried samples involved dispersing a small amount of solid in ethanol followed by sonication. A droplet of the suspension was deposited on a suitable substrate (Al stub with carbon tape for SEM and Lacey Cu grid for TEM) and the solvent was left to evaporate.

### Nanoindentation

To ensure reliable results from nanoindentation tests, the specimen surface was flattened by thoroughly polishing with sandpapers and diamond suspensions. All tests were carried out using a KLA iMicro nanoindenter, equipped with a 50 mN force actuator and using a Berkovich tip. Continuous Stiffness Measurements (CSM) were performed, a modulus

(1) $$E^{*}\left(E^{*}=E/\;\;\left(1-\nu^{2}\right)\right)$$

used in place of Young's modulus *E* when the Poisson's ratio *n* is unknown – and Hardness *H* as a function of the indentation depth. 2 sets of 16 indents were performed in different areas, setting 1000 nm as the maximum indentation depth. The tests were carried out using a constant indentation strain rate of 0.1 s^−1^. The max load was held for 1 s before unloading to quantify creep. Upon unloading, the load was held again at 10% of max load for 3 min., in order to quantify thermal drift and correct the recorded value of load and depth. The CSM values of *E* ^*^ and *H* were averaged in the interval 500–1000 nm.

### Gas Adsorption

N_2_ adsorption isotherms at 77 K were carried out using an Anton Paar Nova‐800. Activated samples were submitted to ex situ degassing in a built‐in degasser (110 °C, 20 °C min^−1^, 6 h). Isotherms were collected and evaluated using Kaomi software. Apparent surface areas were calculated from isotherms using the BET equation. Micropore volumes and micropore size distributions (when applicable) were obtained using the Dubinin–Radushkevich (DR) and Dubinin–Astakhov (DA) equations, respectively. Mesopore size distributions were obtained using the Barrett–Joyner–Halenda (BJH) model applied to the desorption branch of the corresponding isotherm down to a relative pressure (*P*/*P*_0_) of 0.2. Total pore volumes were obtained by taking readings at *P*/*P*_0_ = 0.95. While DFT methods might be more accurate than so‐called “classical” methods when analyzing pore size distributions,^[^ ^56^ ^]^ the lack of a suitable kernel consistent with the experimental system made use of the DA and BJH models a more accurate approach to analyze current results. These methods were used to compare data between samples rather than to provide an absolute method for the analysis of micro/mesopore size distribution.

### Hg Porosimetry

Helium pycnometry measurements (real density measurements) were performed in the MicroUltrapycnometer apparatus from Anton‐Paar (Quantachrome). Mercury intrusion porosimetry measurements were taken in a POREMASTER‐60 GT apparatus from Anton‐Paar (Quantachrome). For mercury intrusion porosimetry experiments, samples were introduced in a glass sample holder of known volume (by calibration‐filling with mercury). The cell was evacuated and filled with mercury until electrical contact +0.1 psi was achieved. At this point, the mercury filling stopped completely, and the low‐pressure intrusion measurement started (pneumatic pressure of N_2_). At the end of the low‐pressure intrusion, the cell was recovered from the low‐pressure chamber and weighed to calculate the mass and volume of mercury. The sample cell was then submitted to the high‐pressure chamber in the porosimeter where the intrusion experiment continued up to ≈35 500 psi. Sample volume at any desired pressure (and therefore density) could be calculated by subtracting the intruded volume at any given pressure from the measured volume at fill.

### Dynamic Light Scattering and Zeta Potential

DLS and Zeta potential measurements were carried out using a Malvern Panalytical Zetasizer Nano ZS with a 633 nm He‐Ne Laser. Aliquots were taken directly from reaction mixtures and diluted to maintain the mean count rate in the range of 300–450 kcps. A DT folded capillary cell was used to measure the zeta potential at 25 °C. Refractive indices and densities of solvent mixtures were calculated after Scott and Nikumbh et al.^[^ ^57^ ^]^

### Thermogravimetric Analysis

Thermogravimetric analysis (TGA) was performed on a Mettler Toledo TGA/DSC 2 STAR^e^ system. Samples of 10–20 mg were heated to 700 °C at a rate of 10 °C min^−1^. Measurements were performed under a constant flow (80 mL min^−1^) of air (19–22% O_2_ in N_2_, <10 ppm H_2_O, Air Liquide UK Limited).

### UV–Vis Spectroscopy

Post‐synthetic MOF doping was analyzed by taking advantage of inherent UV absorption by NADH. UV–Vis spectroscopy was performed on a Perkin Elmer Lambda 750 and data were processed using UV WinLab and Origin 2021b. The activated monolith was placed in a small vial containing an aqueous solution of NADH, FDH, and GDH (5 mg each in 20 mL). The vial was placed on an orbital shaker and the solution was analyzed over a period of 6 h. GDH and FDH can be assayed using the production of NADH (340 nm).^[^ ^58^ ^]^

## Conflict of Interest

The authors declare no conflict of interest.

## Author Contributions

A.P. contributed to the conceptualization, methodology, investigation, formal analysis, validation, writing of the original draft, and writing – review and editing, as well as data curation. L.A.A. participated in the investigation, formal analysis, and writing – review, and editing. J.F.C. was involved in the investigation, formal analysis, writing – review, and editing. M.T. contributed to the investigation, formal analysis, writing of the original draft, and writing – review and editing. D.C.A. secured funding, contributed to the methodology and supervision, and participated in writing – review and editing. J.C.T. acquired funding, assisted in the methodology and supervision, performed validation, and contributed to writing – review and editing. Á.B.M. developed the methodology, supervised the study, performed validation, wrote parts of the original draft, contributed to writing – review and editing, and curated data. G.M. obtained funding, managed project administration, and participated in writing – review and editing. A.E.H.W. led the conceptualization, secured funding, managed project administration and resources, supervised the study, performed validation, contributed to writing – review and editing, and curated data.

## Supporting information



Supporting Information

## Data Availability

Detailed data for this paper are available at https://doi.org/10.17863/CAM.116966.
