# Supplementary material for: Understanding Metal–Organic Framework Densification: Solvent Effects and the Growth of Colloidal Primary Nanoparticles in Monolithic ZIF‐8
Source: Small. 2025 Apr 13;21(21):2500510. doi: 10.1002/smll.202500510 (PMC12105442; doi:10.1002/smll.202500510)
Supplement: Supplementary file 1 — Supporting Information [file SMLL-21-2500510-s001.docx]

Electronic Supplementary Information

**Understanding Metal-Organic Framework Densification: Solvent Effects and the Growth of Colloidal Primary Nanoparticles in Monolithic ZIF-8**

*Ayush Pathak, Lana A. Alghamdi, Javier Fernández-Catalá, Michele Tricarico, Diego Cazorla-Amorós, Jin-Chong Tan, Ángel Berenguer-Murcia, Gift Mehlana, Andrew E. H. Wheatley**

A. Pathak, L. A. Alghamdi, A. E. H. Wheatley

Yusuf Hamied Dept. of Chemistry, University of Cambridge, Lensfield Road, Cambridge, CB2 1EW, United Kingdom

E-mail: aehw2@cam.ac.uk

L. A. Alghamdi

Centre of Excellence for Nanomaterials for Clean Energy Applications, King Abdulaziz City for Science and Technology, P.O. Box 6086, Riyadh 11442, Saudi Arabia

J. Fernández-Catalá, D. Cazorla-Amorós, Á. Berenguer-Murcia

Dept. of Inorganic Chemistry and Materials Institute, Universidad de Alicante, Apdo. 99, Alicante, Spain

M. Tricarico, J-C. Tan

Multifunctional Materials & Composites (MMC) Lab., Dept. of Engineering Science, University of Oxford, Parks Road, Oxford, OX1 3PJ, United Kingdom

G. Mehlana

Dept. of Chemical Sciences, Faculty of Science and Technology, Midlands State University, P Bag 9055, Senga Road, Gweru, Zimbabwe

1 Sample Optical Images S2

2 Powder X-ray Diffraction S3

3 Attenuated Total Reflection Fourier Transform Infrared Spectroscopy S4

4 Yield and Microanalysis S5

5 Scanning Electron Microscopy S6

6 Transmission Electron Microscopy S10

7 Nanoindentation S18

8 Thermogravimetric Analysis S20

9 BET and Porosimetery S21

10 Dynamic Light Scattering S23

11 ζ-Potential S24

12 Solvent Density Measurements S25

13 Post-synthetic doping S26

14 References S27

# Sample Optical Images


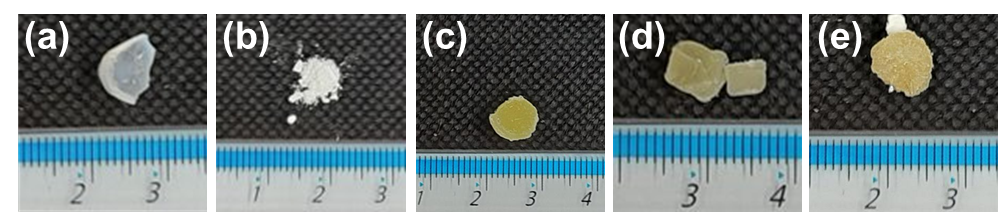


**Figure S1.** Selected samples of ZIF-8 prepared in this work: (a) _mono_ZIF-8 (Main manuscript, Table 4, entry 1, see below); (b) pZIF-8 (entry 2); (c) mZIF-8-R (entry 11); (d)mZIF-8-Δ (entry 12); (e) mZIF-8-CS (entry 13). The white material in (e) is attributed to the exclusion of CaSO_4_ from the adjacent piece of evolving monolith.

# Powder X-ray Diffraction

    
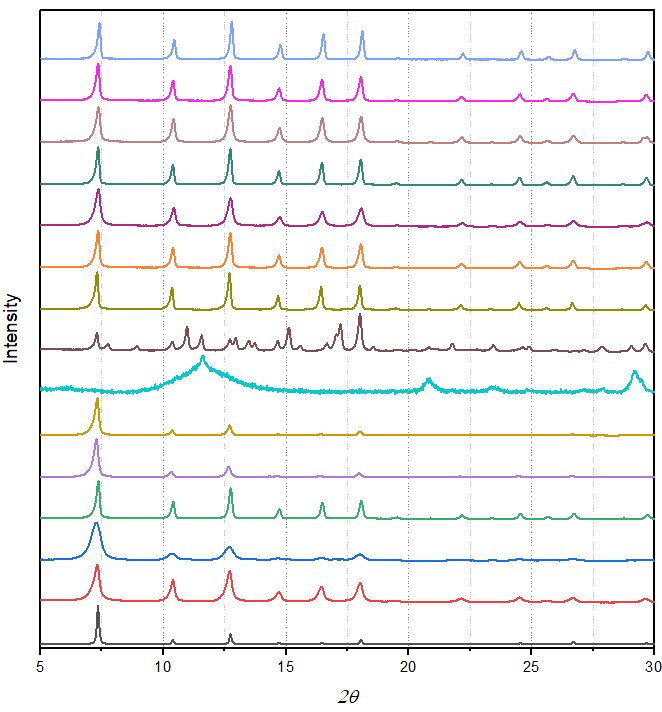


**pZIF-8-h**

**pZIF-8-g**

**pZIF-8-f**

**pZIF-8-e**

**pZIF-8-d**

**pZIF-8-c**

**pZIF-8-b**

**pZIF-8-a**

**Ca residue**

**mZIF-8-CS**

**mZIF-8-Δ**

**mZIF-8-R**

**_mono_ZIF-8**

**pZIF-8**

**simZIF-8**

**Figure S2.** PXRD data for samples reported in main manuscript Table 4, with the simulated pattern for ZIF-8 (bottom).^1,2^


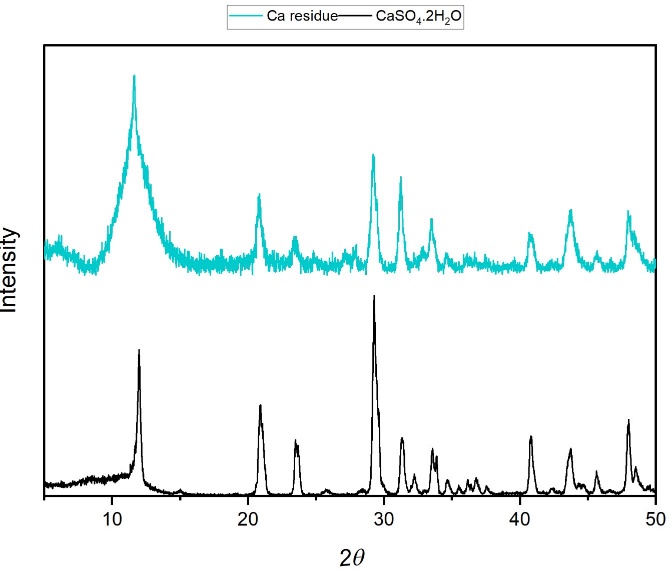


**Figure S3.** PXRD data for (**—**) Ca residue and (**—**) a commercial sample of CaSO_4_×2H_2_O. Major matching peaks are at 11°, 21°, 23°, 29°, 31°, 33°, 40°, 43° and 47°.^2^

# Attenuated Total Reflection Fourier Transform Infrared Spectroscopy


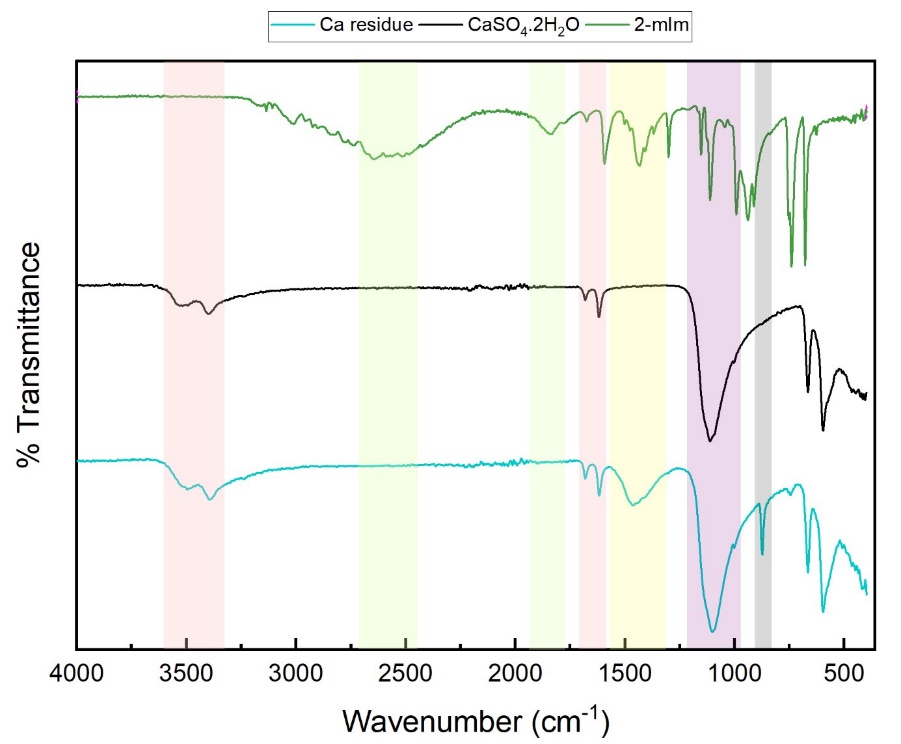


**SO_4_^2-^**

**NO_3_^-^**

**NO_3_^-^**

**-NH···N**

**-NH**

**H_2_O**

**H_2_O**

**Figure S4.** ATR FTIR data for (**—**) Ca residue, (**—**) CaSO_4_×2H_2_O and (**—**) 2-mIm. Calcium sulfate dihydrate presents bands at 3600-3300 cm^-1^ and 1700-1600 cm^-1^ corresponding to the stretching frequency of -OH and deformation vibrations from H_2_O respectively and broad bands at 1200-1000 cm^-1^ and 670-600 cm^-1^ correspond to triple degenerate vibrations of sulfate ion.^2^ 2750-2450 cm^-1^ corresponds to the -NH stretching and 1950-1780 cm^-1^ to -NH···N peak from 2-mIm. Band at 1550-1350 cm^-1^ and peak at 800 cm^-1^ in Ca residue which doesn’t match with either precursors can be attributed to the residual NO_3_^-^ from zinc precursor.^3^

# Yield and Microanalysis

**Table S1.** Yields and compositional analyses for activated pZIF-8 and mZIF-8 prepared using 15% v/v EtOH−H_2_O conditions. *Monolith. ^§^White deposit.

| **Entry** | **Yield (%)** | **Zn (at%)** | **C (at%)** | **H (at%)** | **N (at%)** | **Ca (at%)** |
| --- | --- | --- | --- | --- | --- | --- |
| Theoretical | – | 28.7 | 42.2 | 4.4 | 24.7 | – |
| pZIF-8 | 85 | 29.5 | 41.6 | 4.3 | 23.9 | – |
| mZIF-8-R | 79 | 27.2 | 39.2 | 4.1 | 21.7 | – |
| mZIF-8-Δ | 83 | 27.9 | 39.8 | 3.9 | 22.3 | – |
| mZIF-8-CS* | 87 | 26.4 | 39.4 | 4.0 | 22.3 | 3.5 |
| mZIF-8-CS^§^ | – | 1.3 | 18.2 | 0.9 | 4.6 | 35.6 |

# Scanning Electron Microscopy

**(E)**


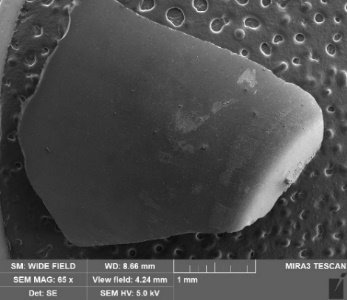

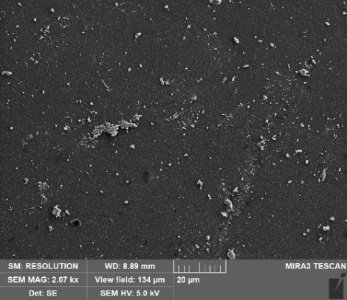


**(C)**

**(A)**

**(B)**

**(D)**


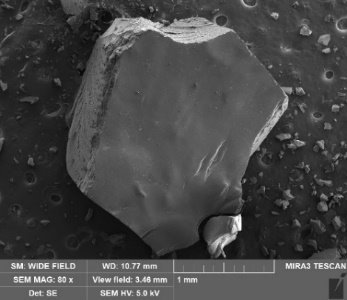

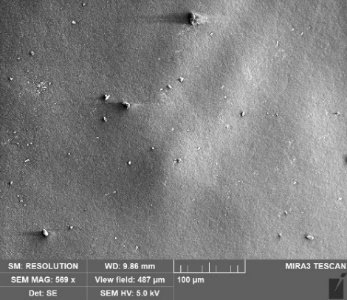


**(F)**


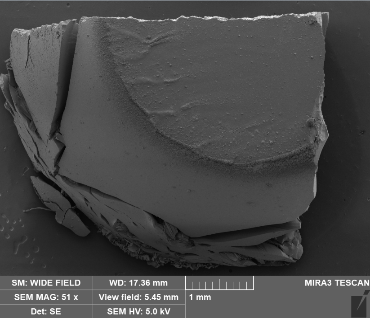

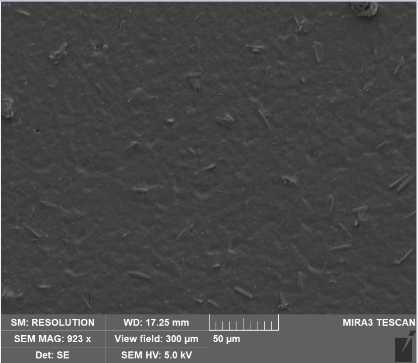


**Figure S5.** Representative low and high magnification SEM images of samples (A, B) _mono_ZIF-8 (see main manuscript Table 4, entry 1), (C, D) pZIF-8 (entry 3) and (E, F) mZIF-8-Δ (entry 12). Scale bars: 1 mm (A, C, E), 20 μm (B), 100 μm (D), 50 μm (F).

**(C)**

**(A)**

**(D)**

**(B)**


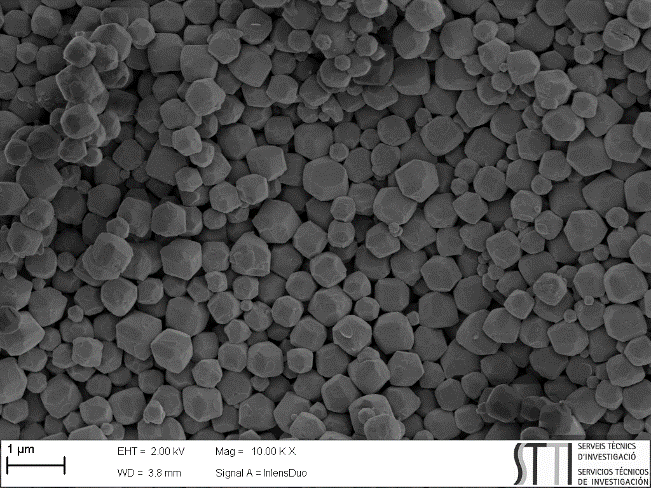

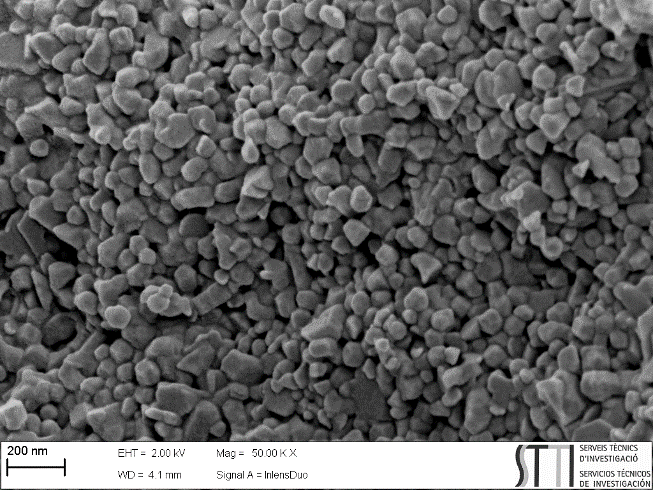

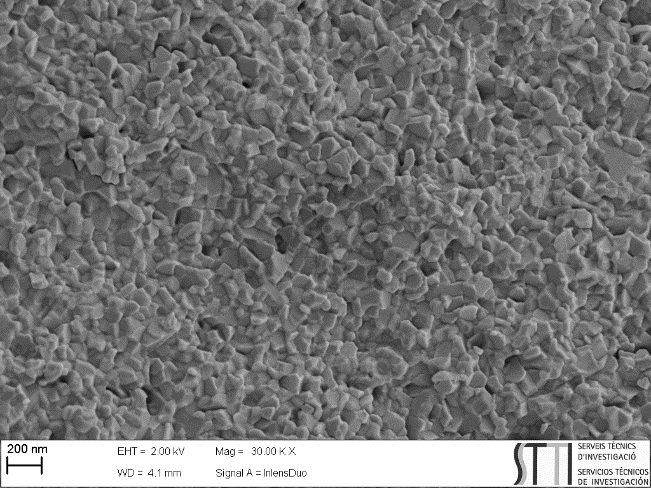

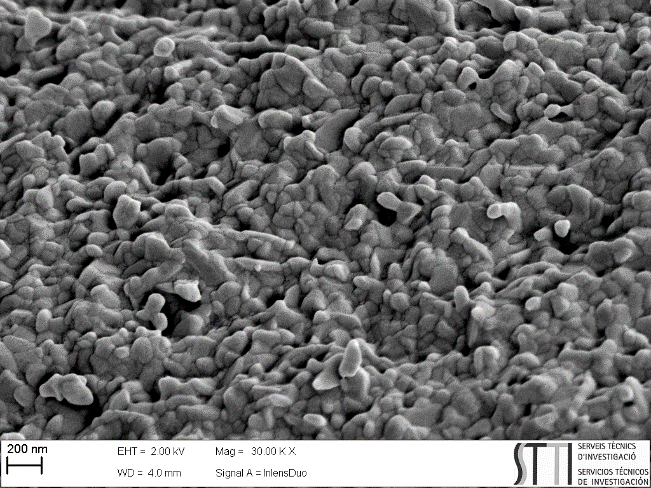


***Figure S6.*** *High magnification SEM images of representative samples: (A) pZIF-8 (Table 4 entry 3), (B) mZIF-8-R (entry 11), (C) mZIF-8-Δ (entry 12) and (D) mZIF-8-CS (entry 13). Scale bars: 1 μm (A), 200nm (B, C, D).*


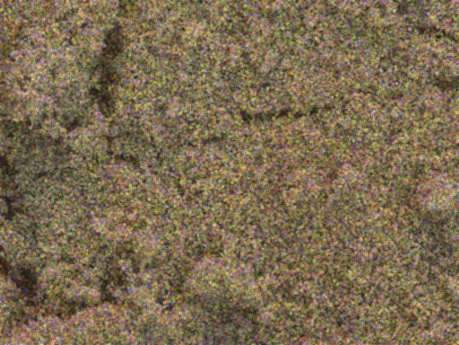


**(D)**

**(C)**

**(A)**

**(B)**

| **Element** | **Wt.%** |
| --- | --- |
| **C** | 38.25 ± 0.08 |
| **N** | 24.38 ± 0.16 |
| **Zn** | 25.54 ± 0.15 |

***Figure S7.*** *SEM-EDS maps of pZIF-8: overlapping elemental map (A), carbon (B), nitrogen (C) and zinc (D). Scale bar: 10 μm. Table showing respective wt.% of carbon, nitrogen and zinc.*


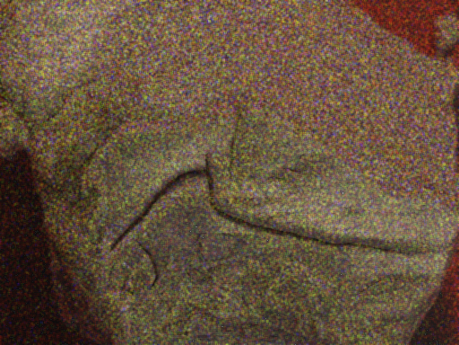


**(D)**

**(B)**

**(A)**

**(C)**

| **Element** | **Wt.%** |
| --- | --- |
| **C** | 40.29 ± 0.04 |
| **N** | 25.75 ± 0.09 |
| **Zn** | 19.36 ± 0.07 |

***Figure S8.*** *SEM-EDS maps of mZIF-8-R: overlapping elemental map (A), carbon (B), nitrogen (C) and zinc (D). Scale bar: 10 μm. Table showing respective wt.% of carbon, nitrogen and zinc.*


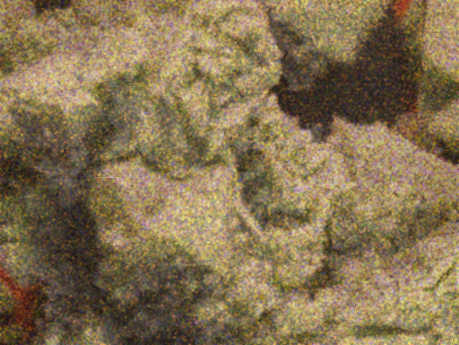


**(D)**

**(C)**

**(B)**

**(A)**

| **Element** | **Wt.%** |
| --- | --- |
| **C** | 39.37 ± 0.02 |
| **N** | 29.13 ± 0.05 |
| **Zn** | 21.41 ± 0.04 |

***Figure S9.*** *SEM-EDS maps of mZIF-8-Δ: overlapping elemental map (A), carbon (B), nitrogen (C) and zinc (D). Scale bar: 10 μm. Table showing respective wt.% of carbon, nitrogen and zinc.*


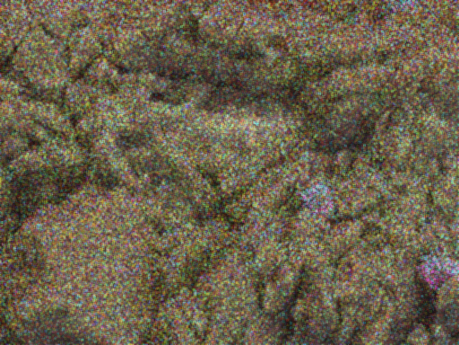


**(E)**

**(F)**

**(A)**

**(B)**

**(C)**

**(D)**

| **Element** | **Wt.%** |
| --- | --- |
| **C** | 40.95 ± 0.04 |
| **N** | 23.85 ± 0.09 |
| **O** | 11.77 ± 0.05 |
| **Ca** | 0.42 ± 0.02 |
| **Zn** | 16.06 ± 0.07 |

***Figure S10.*** *SEM-EDS maps of mZIF-8-CS: overlapping elemental map (A), carbon (B), nitrogen (C), zinc (D), oxygen (E) and calcium (F). Scale bar: 10 μm. Table showing respective wt.% of carbon, nitrogen, oxygen, calcium and zinc.*

# Transmission Electron Microscopy


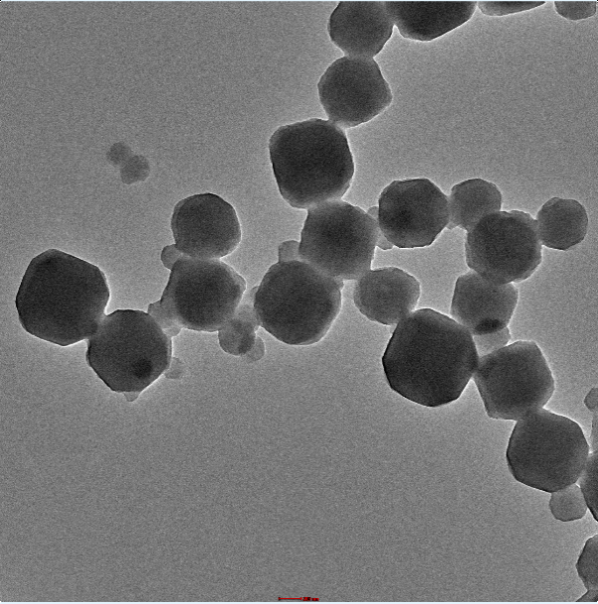

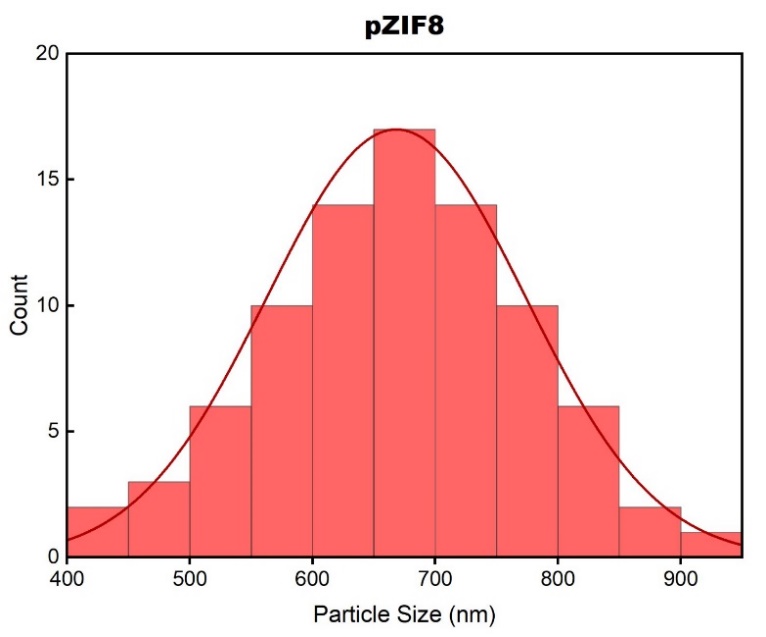


**pZIF-8 – unaged 669 (±106) nm**

**(A)**


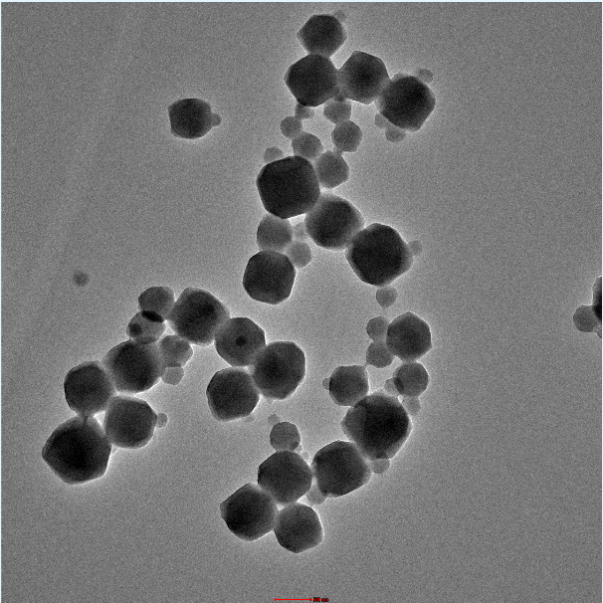

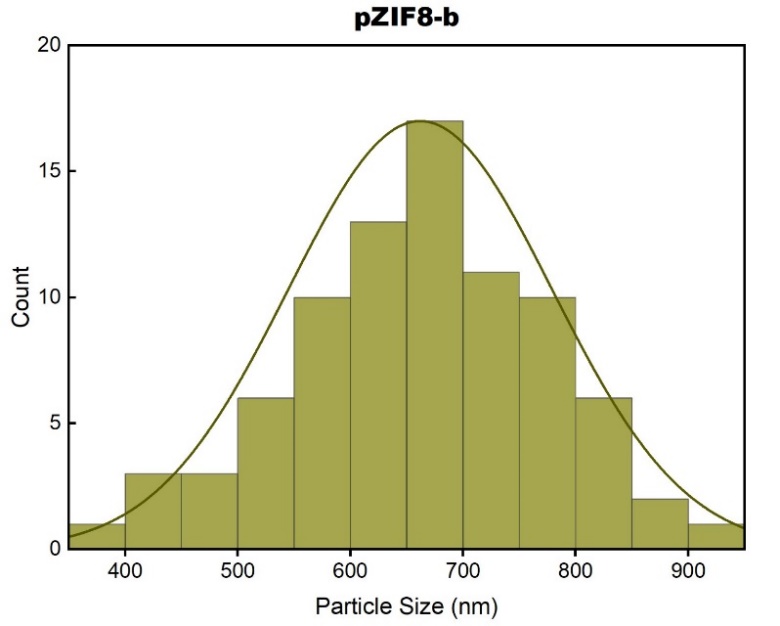


**pZIF-8-b – 3 h aged 661 (±118) nm**

**(B)**


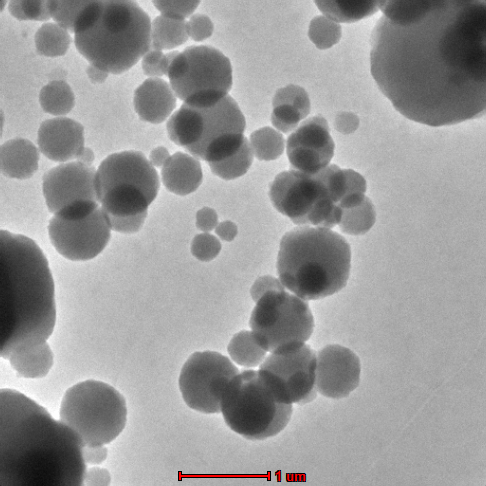

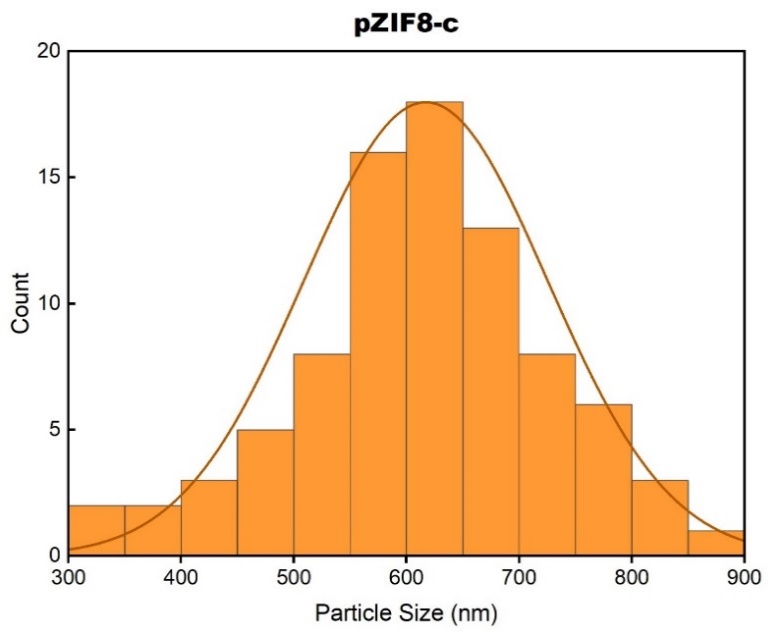


**pZIF-8-c – 24 h aged 618 (±101) nm**

**(C)**


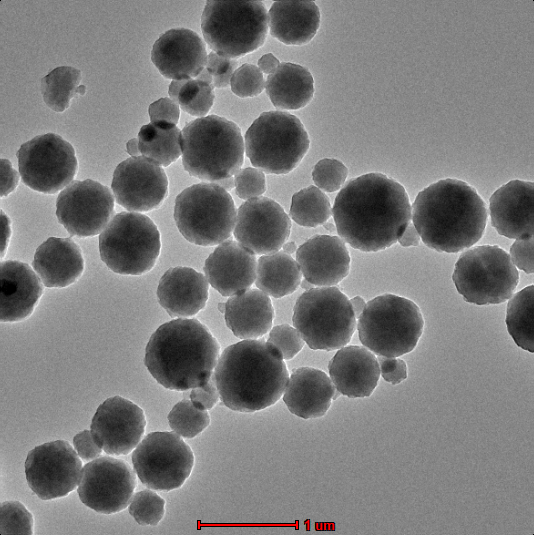

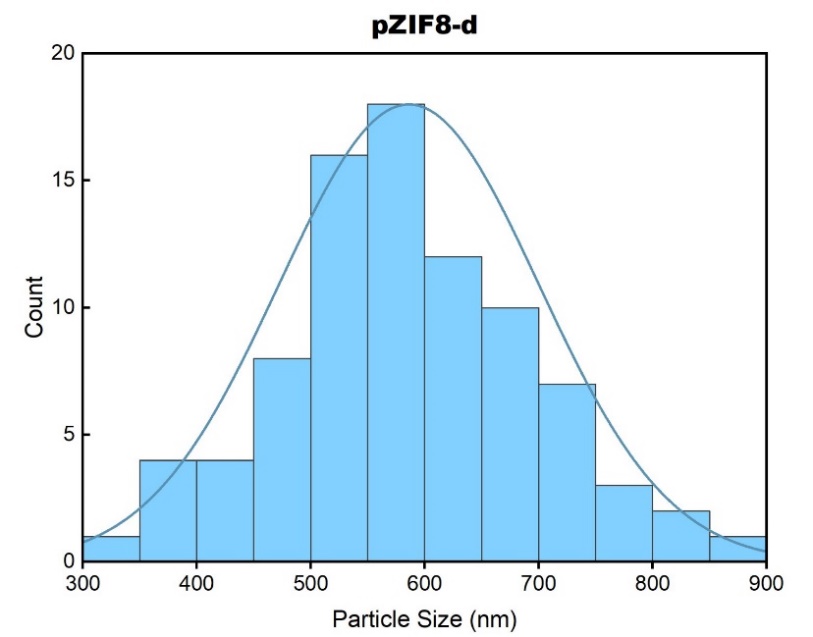


**(D)**

**pZIF-8-d – 48 h aged 586 (±115) nm**


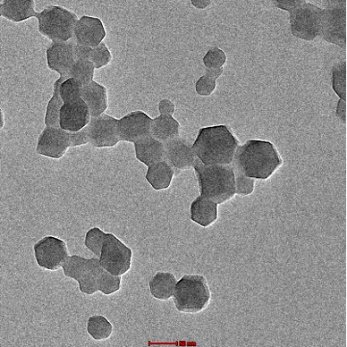

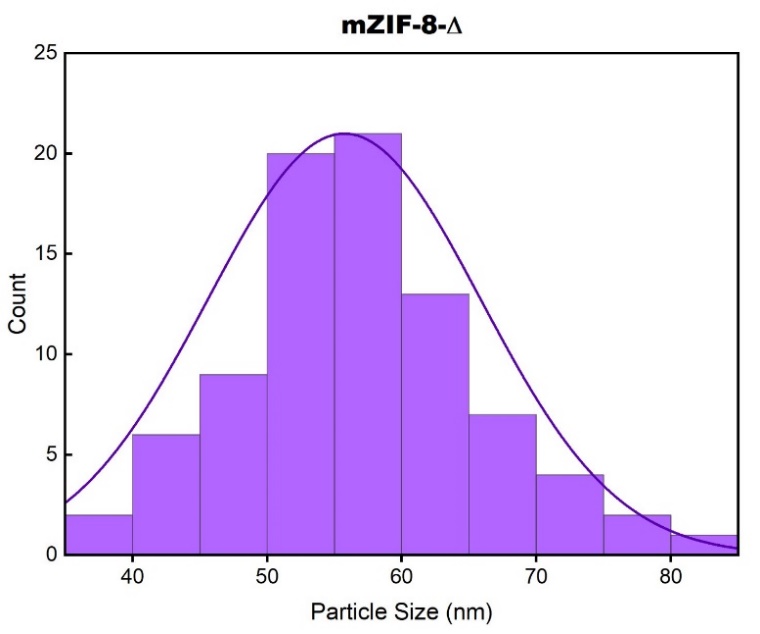


**mZIF-8-Δ – 55 (±10) nm**

**(E)**

**Figure S11.** Representative TEM images and particle size distributions (N = 100) for (A) pZIF-8 (see main manuscript Table 4, entry 3); (B) pZIF-8-b (entry 4, sol aged for 3 h); (C) pZIF-8-c (entry 5, sol aged for 24 h); (D) pZIF-8-d (entry 6, sol aged for 48 h); (E) mZIF-8-Δ (entry 12, 50 ℃ for 96 h). Scale bars: 200 nm (A), 500 nm (B) 1 μm (C, D), 50 nm (E).


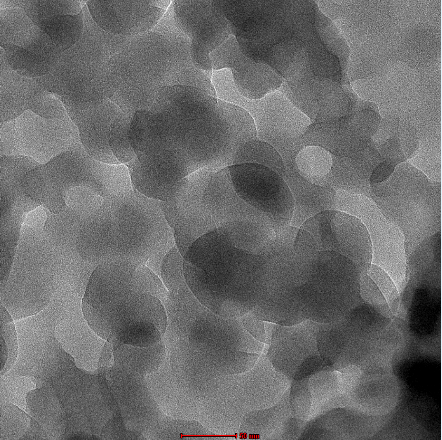

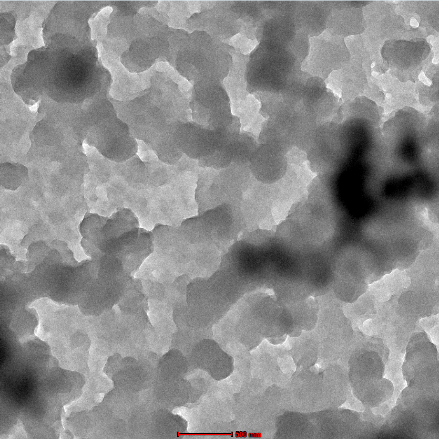


**(B)**

**(A)**

**(D)**

**(C)**


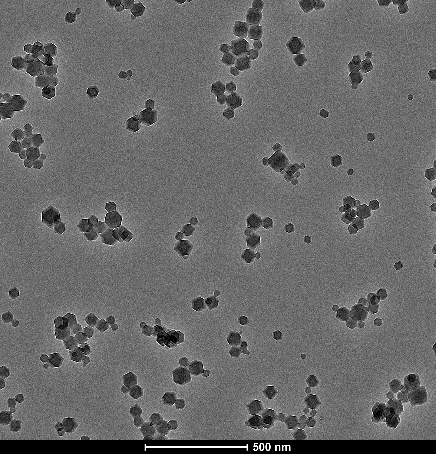

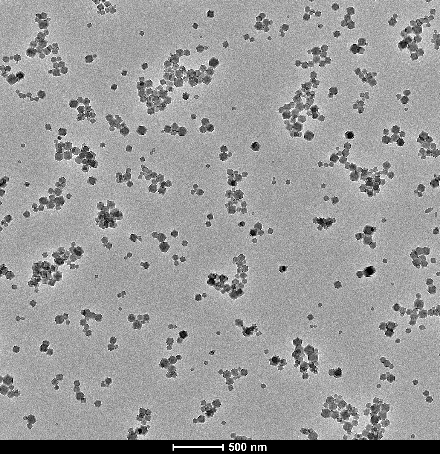


**Figure S12.** Representative TEM images of aliquots of mZIF-8-R reaction mixture (ambient temperature reaction) taken at 15 min (A), 72 h (B) and 120 h (C, D), revealing the rapid emergence of primary nanocrystallites compatible with monolith formation despite gelation taking ~120 h. Scale bars: 50 nm (A), 200 nm (B) and 500 nm (C, D).


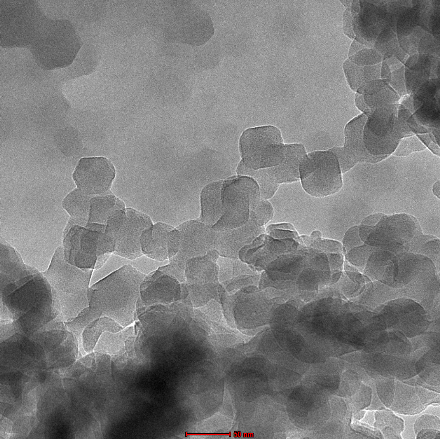

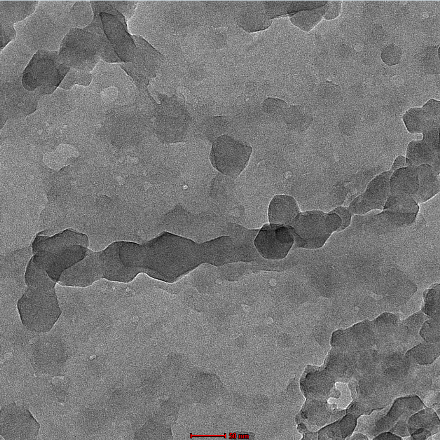


**(B)**

**(A)**

**(D)**

**(C)**


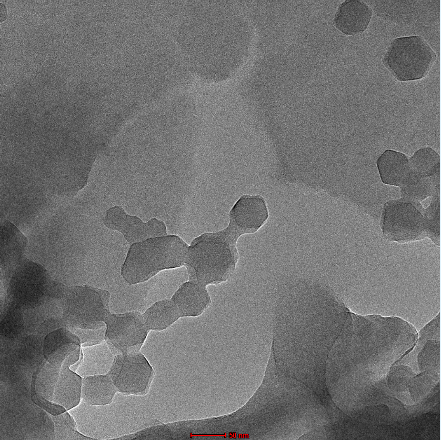

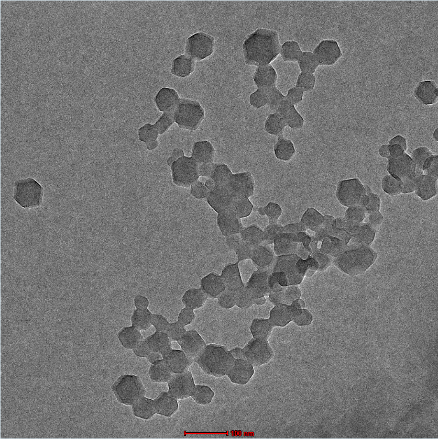


**(F)**

**(E)**


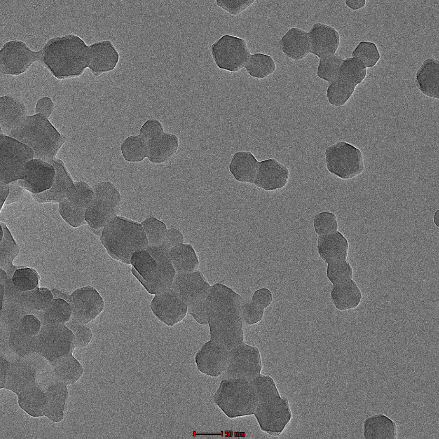

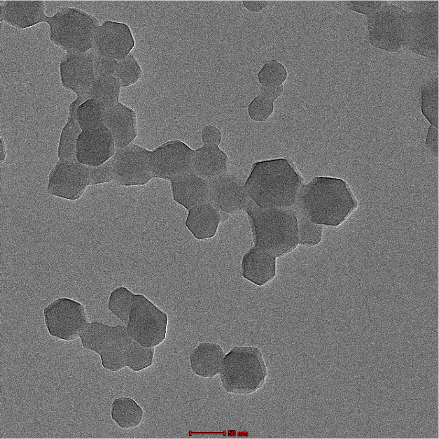


**(H)**

**(G)**


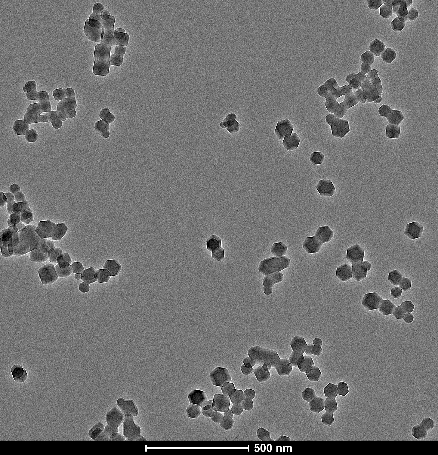

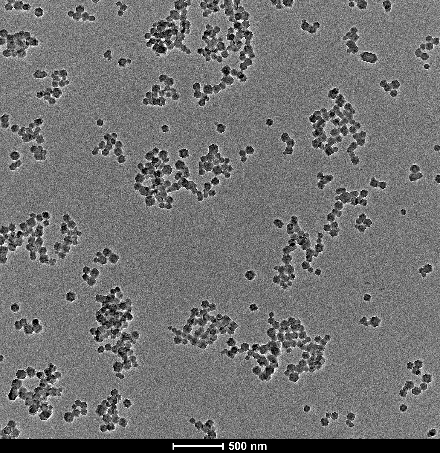


**Figure S13.** Representative TEM images of aliquots of mZIF-8-Δ reaction mixture (50 ℃ reaction) taken at 1 h (A, B), 24 h (C, D), and 96 h (E-H), revealing the rapid emergence of primary nanocrystallites compatible with monolith formation despite gelation taking the full 96 h. Scale bars: 50 nm (A, B, C, E, F), 100 nm (D), 500 nm (G, H).

**(C)**

**(A)**

**(B)**


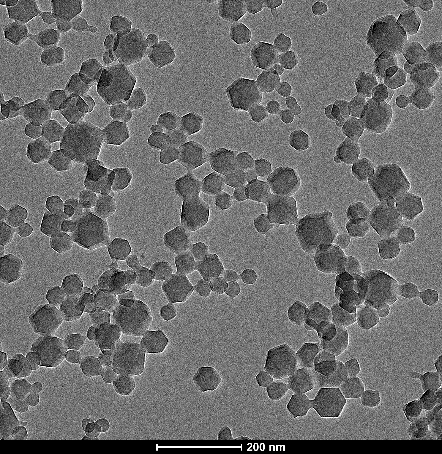

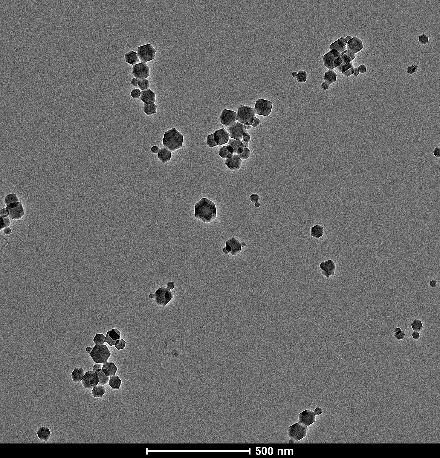

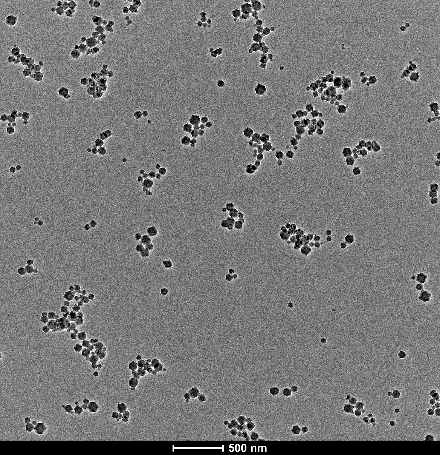


***Figure S14.*** *Representative TEM images of aliquots of mZIF-8-CS reaction mixture (ambient temperature reaction incorporating CaSO_4_) taken after 72 h (A-C) displaying primary nanocrystallites compatible with monolith formation despite gelation taking the full 72 h. Scale bars: 100 nm (A), 250 nm (B), 500 nm (C).*









**(B)**

**(F)**

**(D)**

**(C)**

**(A)**









**(G)**

**(E)**









**(I)**

**(H)**









**(L)**

**(K)**

**(J)**

***Figure S15.*** *Representative TEM images of MOFs after the complete drying of gel followed by activation in a vacuum oven. pZIF-8 (A, B, C), mZIF-8-R (D, E, F), mZIF-8-Δ (G, H, I) and mZIF-8-CS (J, K, L). Scale bars; 5 μm (A, D, G, J), 200 nm (B, C, I, K) and 100 nm (E, F, H, L).*


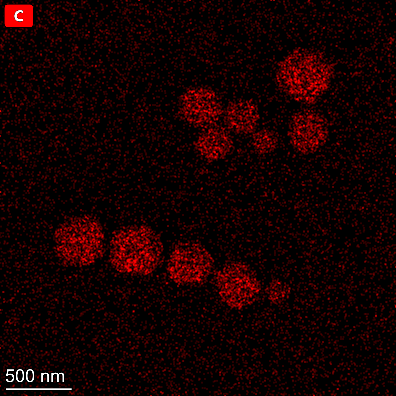

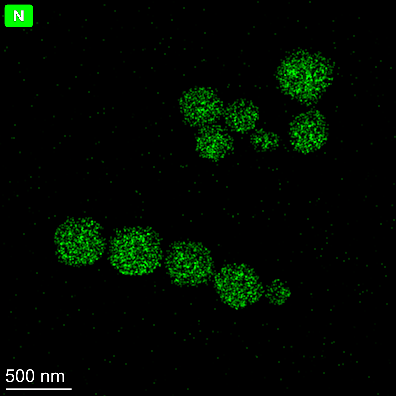

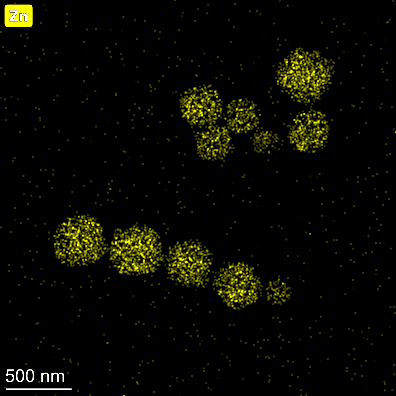

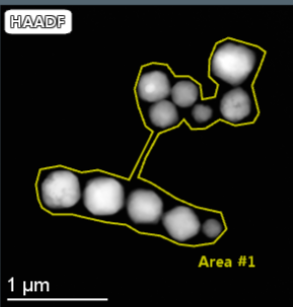


| **Element** | **Wt.%** |
| --- | --- |
| **C** | 48.9 ± 2.4 |
| **N** | 26.0 ± 3.6 |
| **Zn** | 25.2 ± 2.5 |

***Figure S16*.** *STEM-EDS maps of pZIF-8: carbon (red), nitrogen (green), zinc (yellow), HAADF (right) with highlighted area (yellow border) selected for EDS analysis. Scale bar: 500 nm (elemental maps) and 1 μm (HAADF). Table showing respective wt.% of carbon, nitrogen and zinc.*


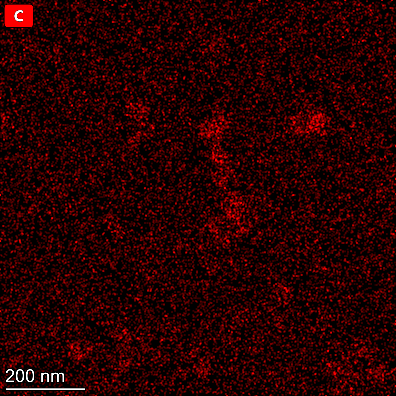

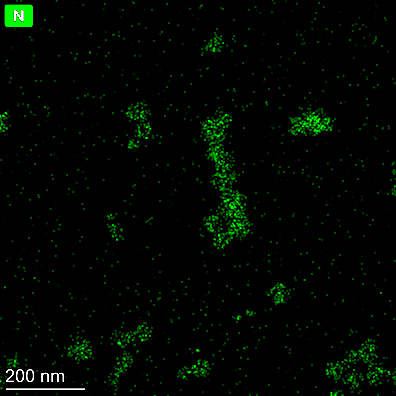

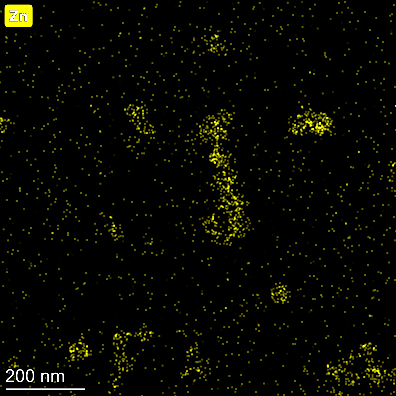

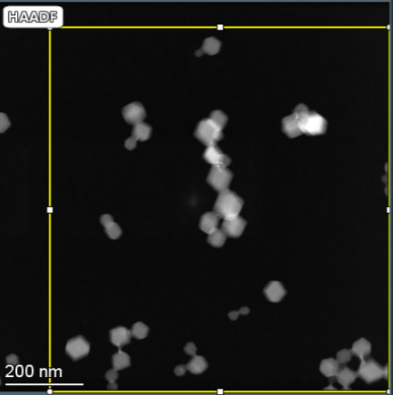


| **Element** | **Wt.%** |
| --- | --- |
| **C** | 90.9 ± 2.1 |
| **N** | 4.9 ± 0.7 |
| **Zn** | 4.2 ± 0.4 |

***Figure S17.*** *STEM-EDS maps of mZIF-8-R: carbon (red), nitrogen (green), zinc (yellow), HAADF (right) with highlighted area (yellow box) selected for EDS analysis. Scale bar: 200 nm. Table showing respective wt.% of carbon, nitrogen and zinc.*


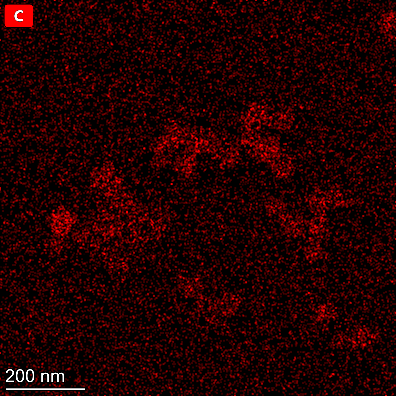

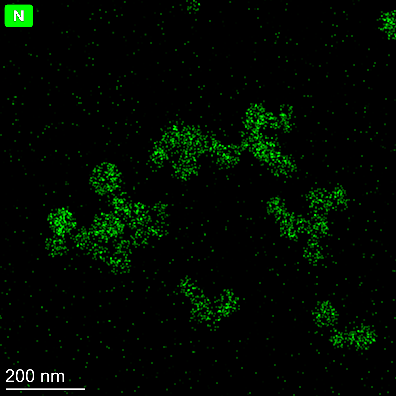

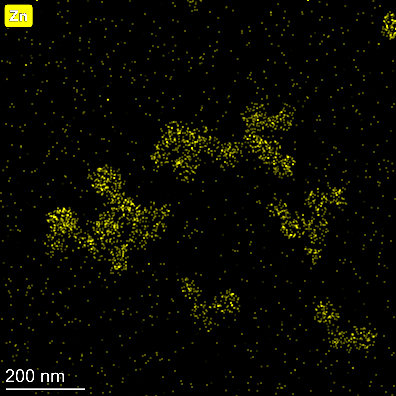

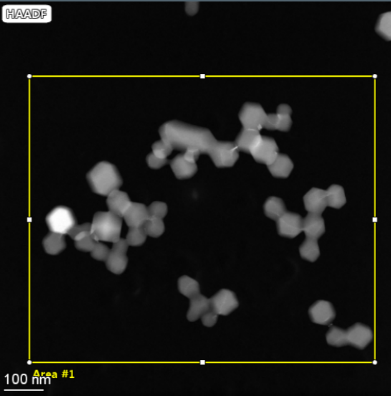


| **Element** | **Wt.%** |
| --- | --- |
| **C** | 79.3 ± 2.0 |
| **N** | 11.2 ± 1.7 |
| **Zn** | 9.6 ± 1.8 |

***Figure S18.*** *STEM-EDS maps of mZIF-8-Δ: carbon (red), nitrogen (green), zinc (yellow), HAADF (right) with highlighted area (yellow box) selected for EDS analysis. Scale bar: 200 nm (elemental maps) and 100 nm (HAADF). Table showing respective wt.% of carbon, nitrogen and zinc.*


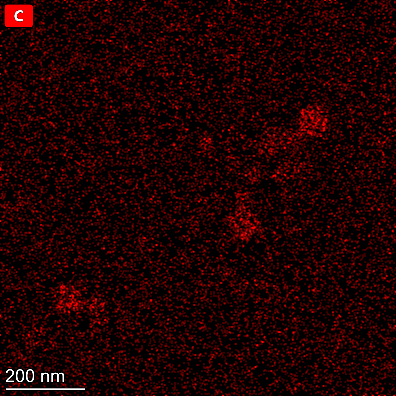

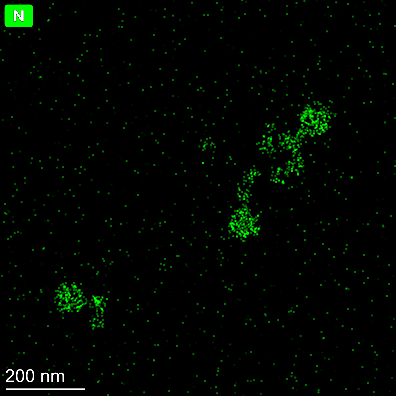

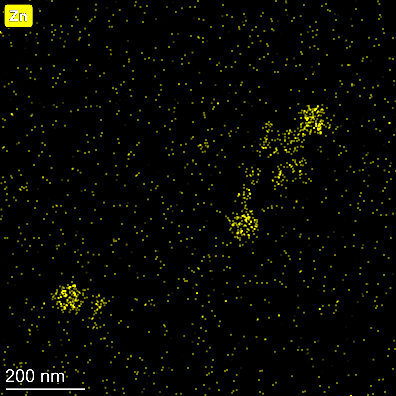

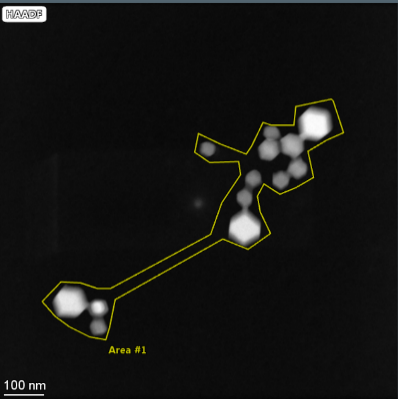


| **Element** | **Wt.%** |
| --- | --- |
| **C** | 69.7 ± 2.2 |
| **N** | 17.2 ± 2.5 |
| **Zn** | 13.1 ± 1.4 |

***Figure S19.*** *STEM-EDS maps of mZIF-8-CS: carbon (red), nitrogen (green), zinc (yellow), HAADF (right) with highlighted area (yellow border) selected for EDS analysis. Scale bar: 200 nm (elemental maps) and 100 nm (HAADF). Table showing respective wt.% of carbon, nitrogen and zinc.*

# Nanoindentation


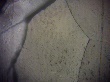

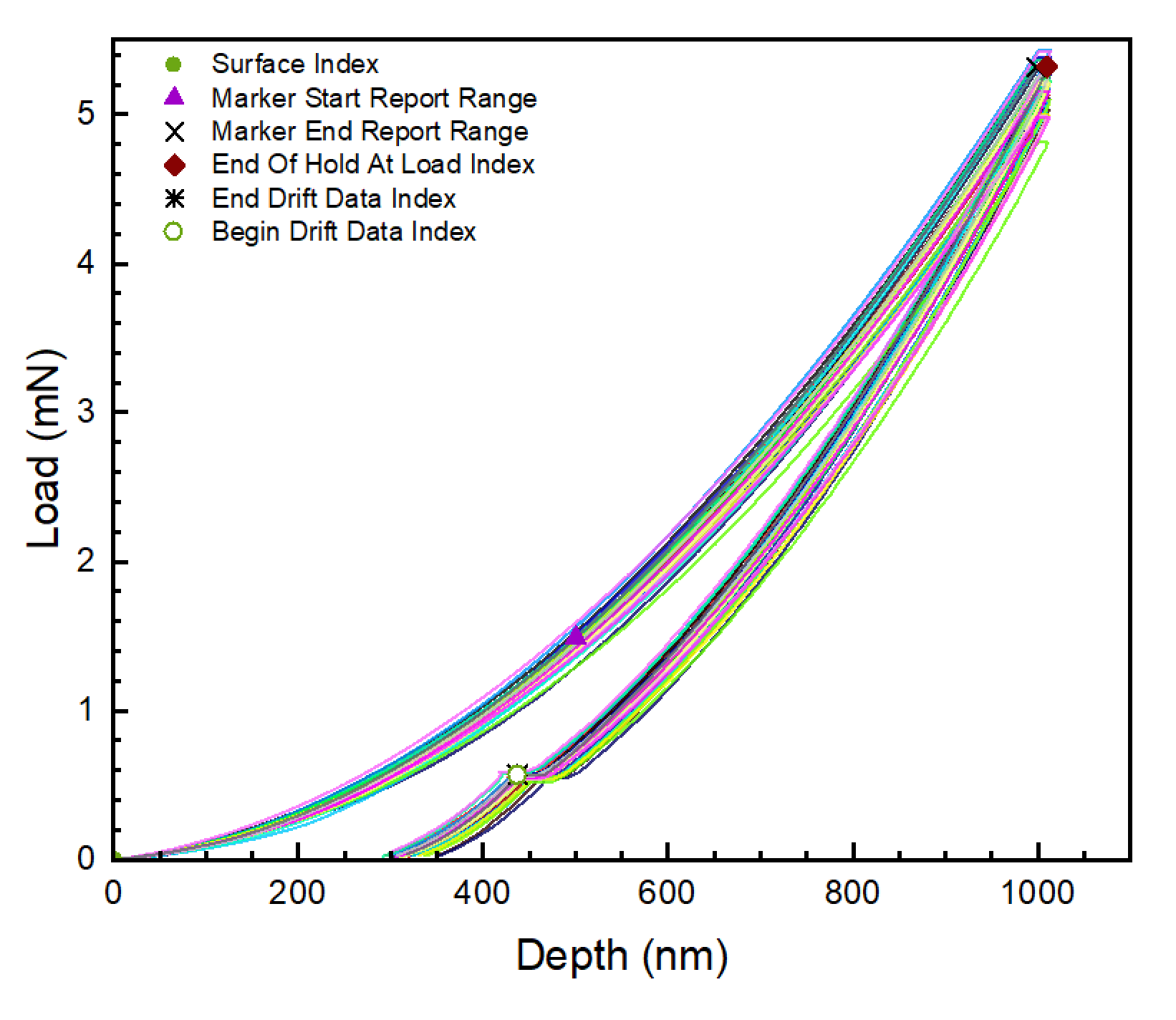


**Figure S20.** Load-displacement (P‑h) nanoindentation data for mZIF-8-Δ. 2 sets of 16 indents were performed in two different areas, setting the maximum indentation depth to 1000 nm. The highly reproducible P‑h data reflect the homogeneity of the sample tested. Inset: Optical microscopy image of the tested surface.


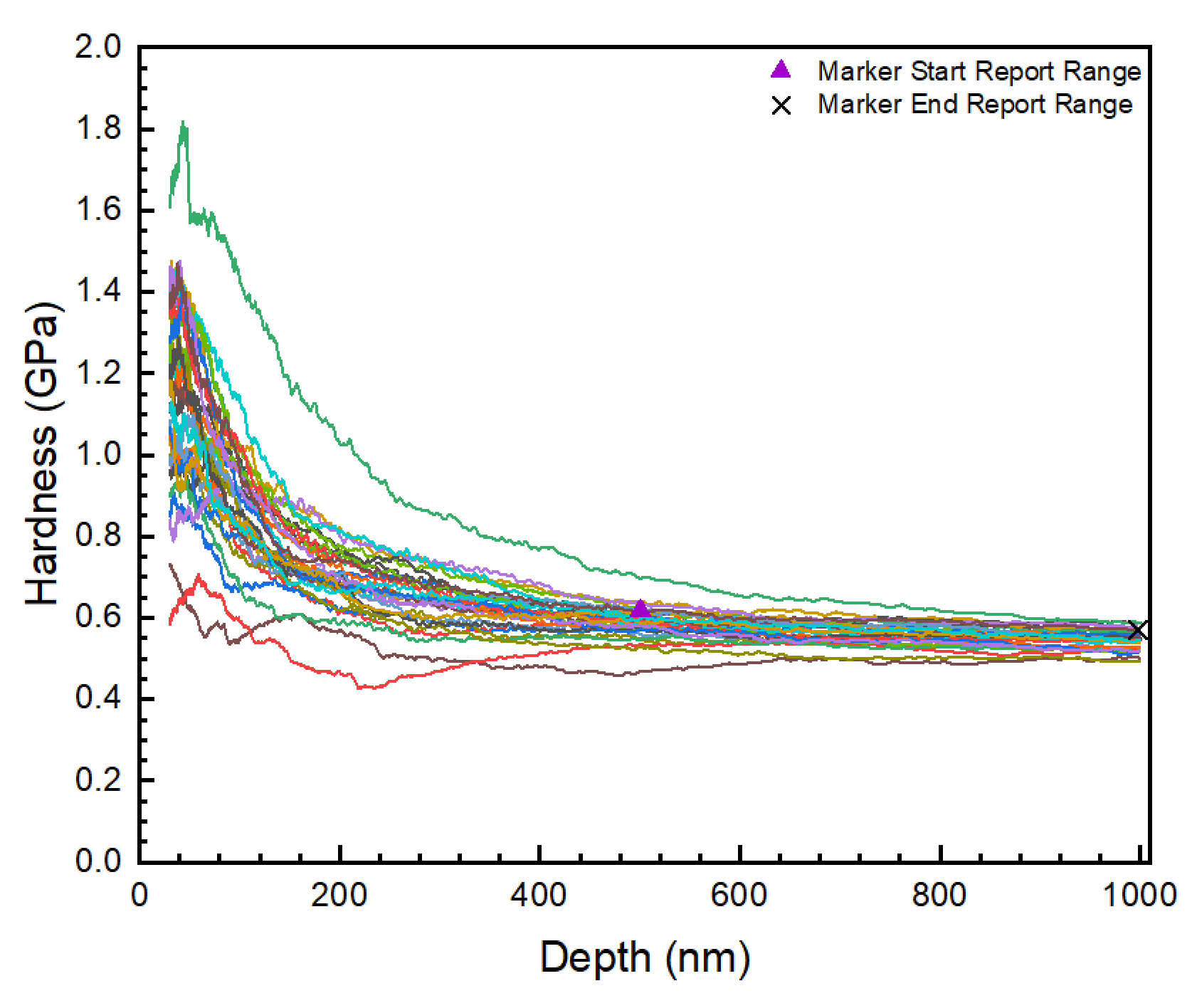


**Figure S21.** Hardness of mZIF-8-Δ plotted as a function of indentation depth. 4 sets of 16 indents were performed. Averaged hardness was determined using data collected over the 500-1000 nm indentation depth range, yielding 0.56 ± 0.03 GPa.


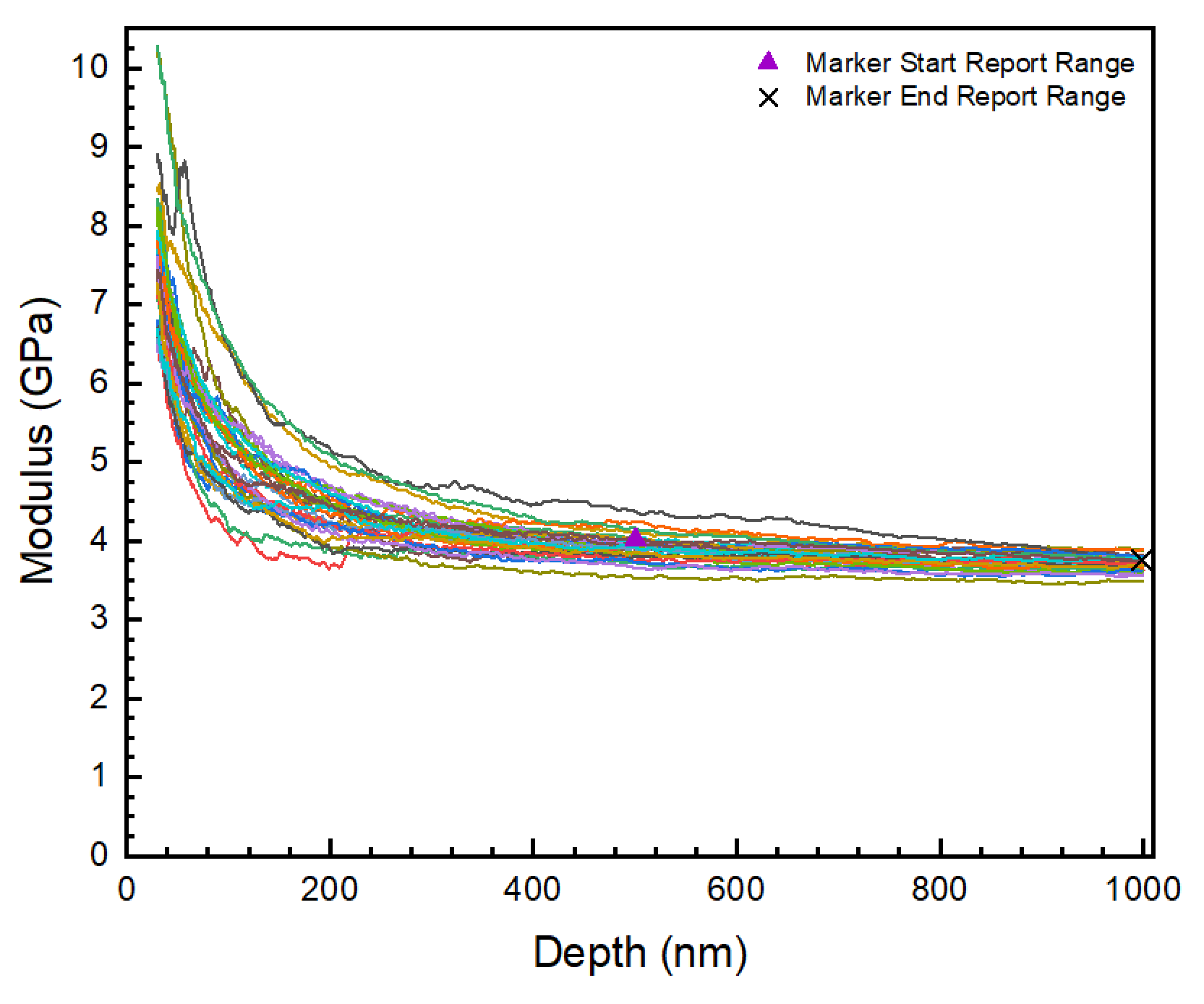


**Figure S22.** Indentation modulus, E*, of mZIF-8-Δ plotted as a function of indentation depth. 4 sets of 16 indents were performed. Averaged indentation modulus was determined using data collected over the 500-1000 nm indentation depth range, yielding 3.8 ± 0.1 GPa.

# Thermogravimetric Analysis


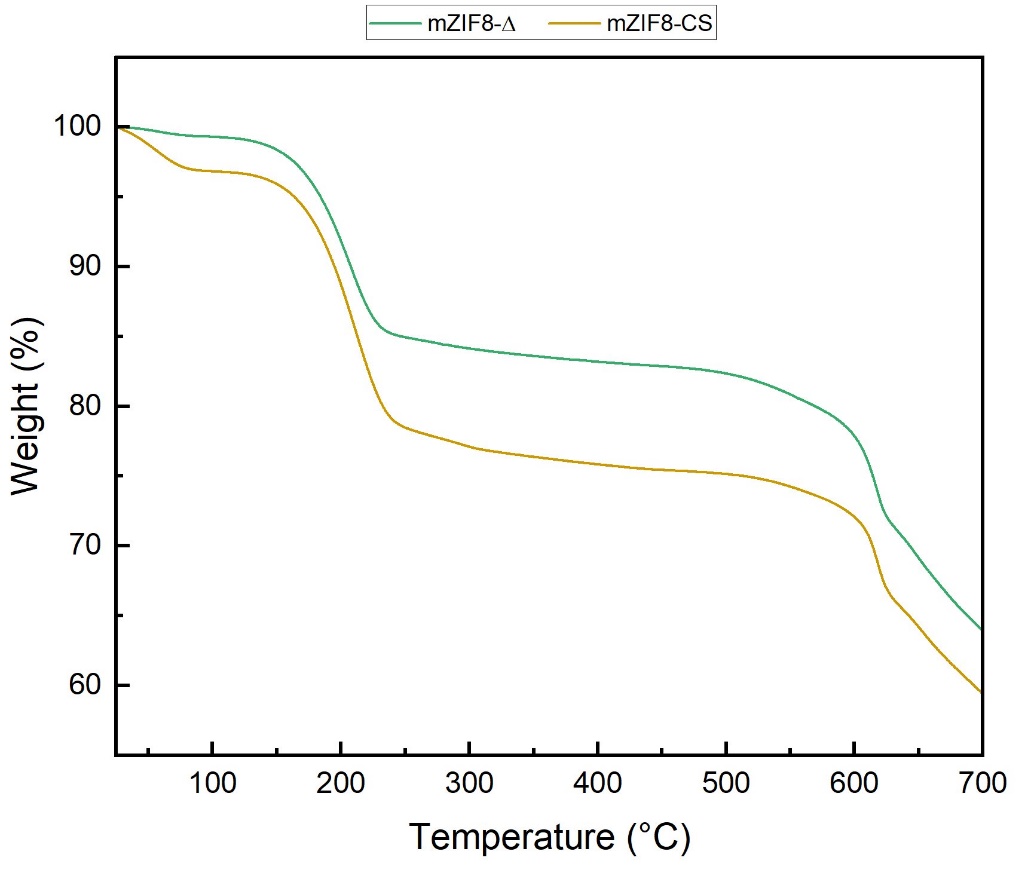


**Figure S23.** Thermogravimetric analysis of mZIF-8-Δ (**—**), and mZIF-8-CS (**—**).

# BET and Porosimetery

 
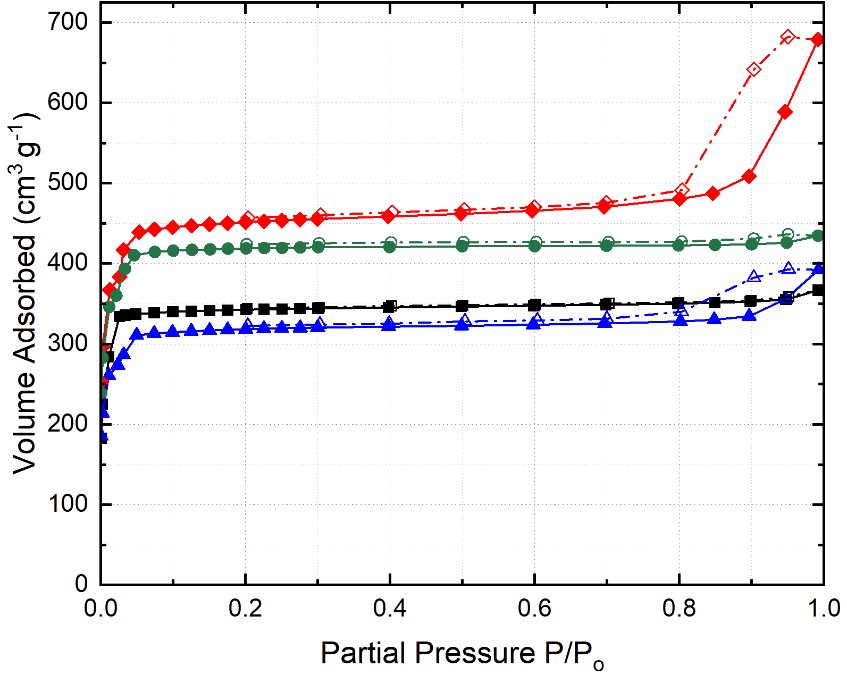


**Figure S24.** N_2_ desorption isotherms for selected samples prepared in this work. Adsorption (coloured marker) and desorption (hollow marker) N_2_ isotherms collected in the range 0-1 bar at 77 K. pZIF-8 (◼ads, **□**des). mZIF-8-R (▲ads, **△**des). mZIF-8-Δ (◆ads, **◇**des). mZIF-8-CS (●ads, 🞅des).


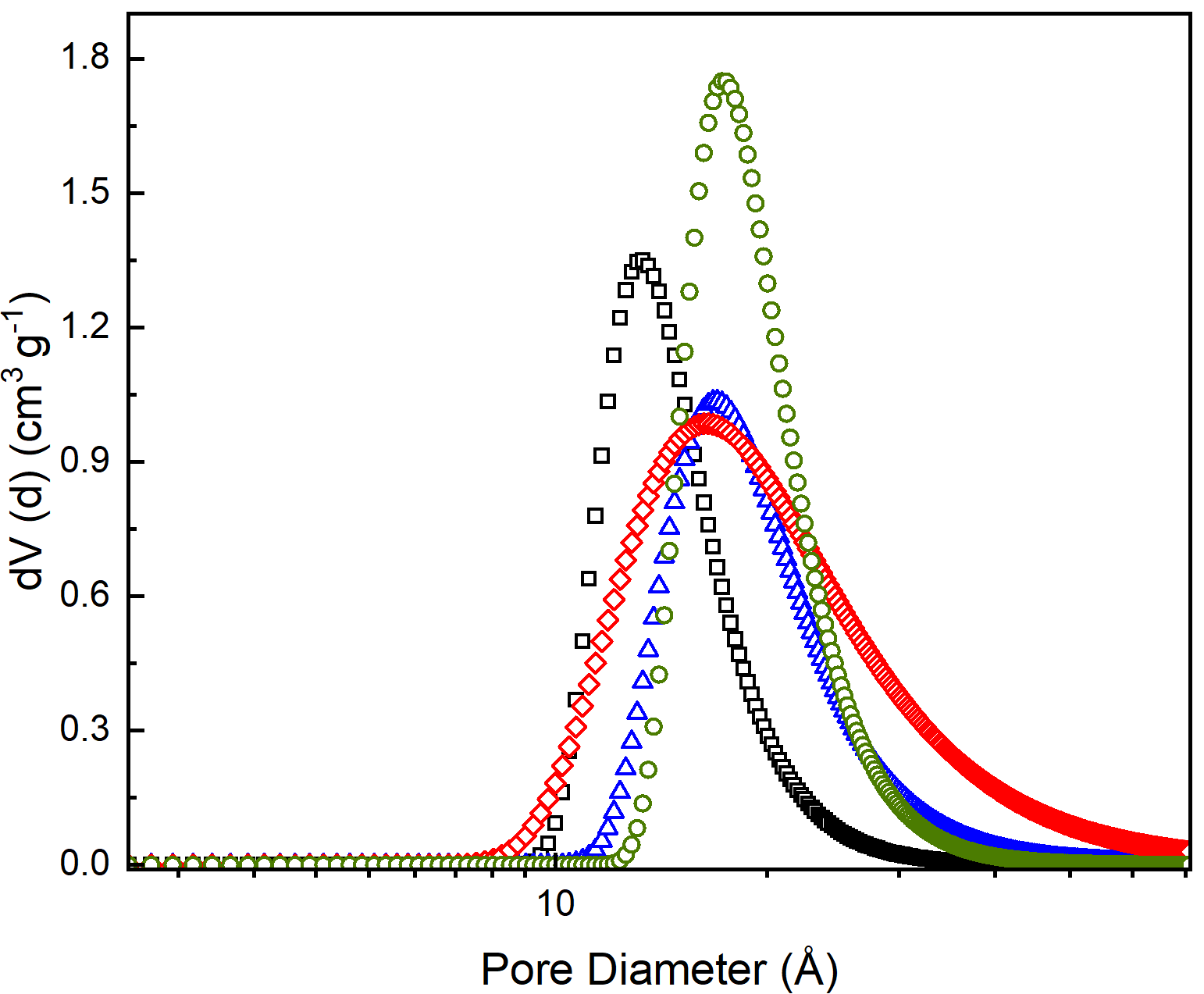


**Figure S25.** Pore size distributions for ZIF-8 prepared in this work. Distribution of micropore diameter as obtained from a DA model analysis of N_2_ isotherm data. **□** pZIF-8; **△** mZIF-8-R; **◇** mZIF-8-Δ; 🞅 mZIF-8-CS.


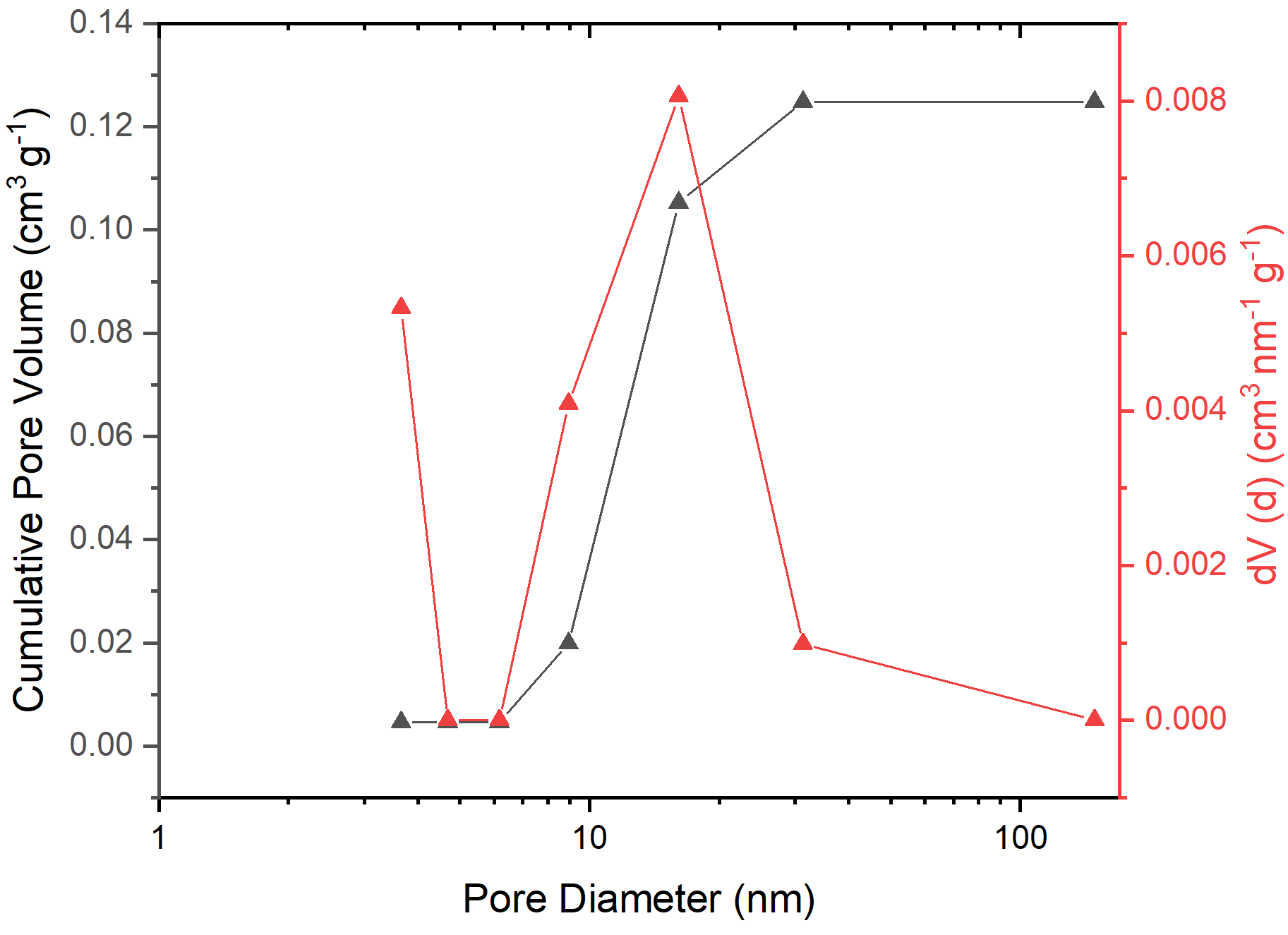

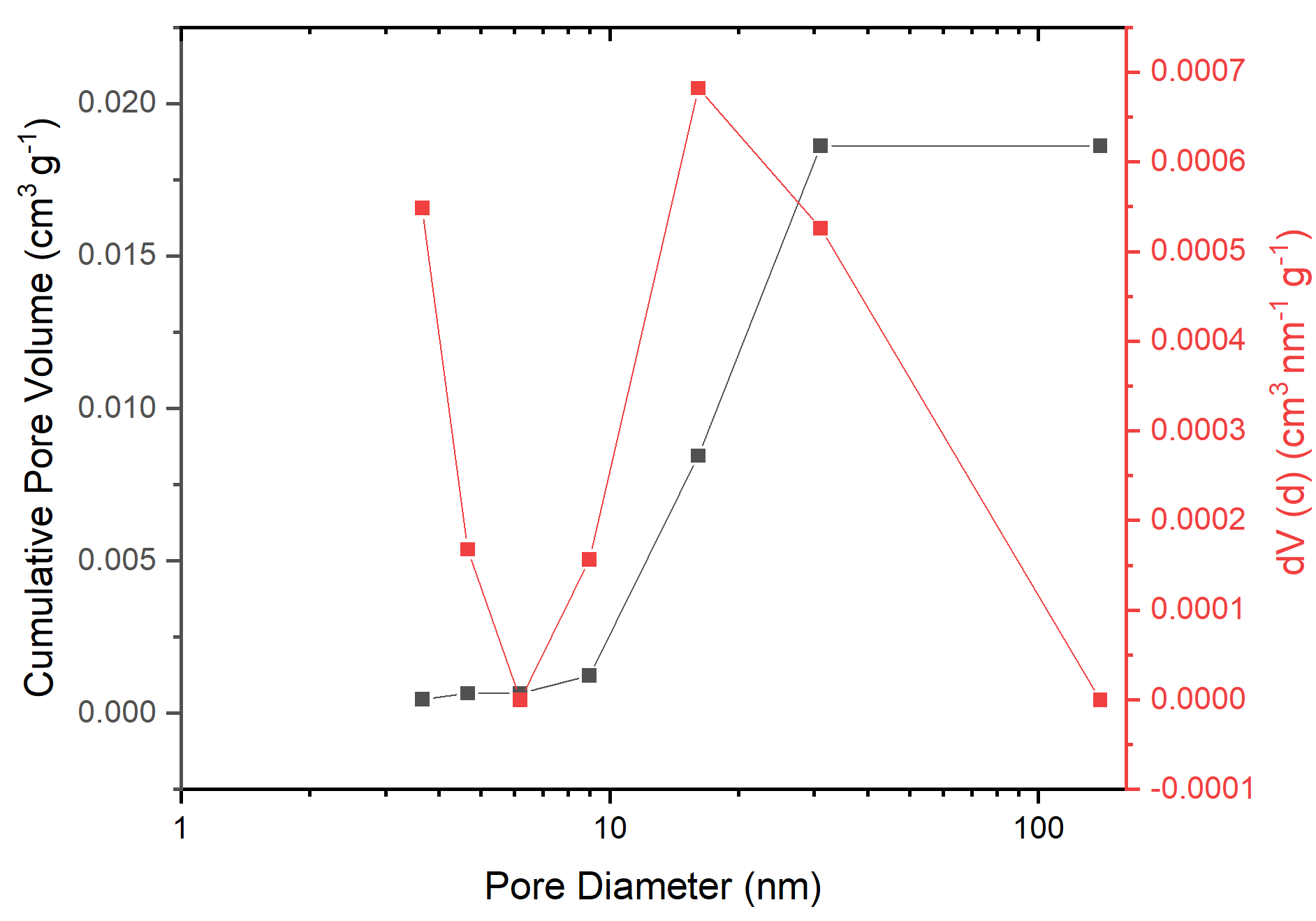


**(A)**

**(B)**

**Figure S26.** Pore size distributions for _mono_ZIF-8 prepared in this work. Distribution of mesopore diameter as obtained from a BJH model analysis of N_2_ isotherm data. (A) mZIF-8-R; (B) mZIF-8-CS.


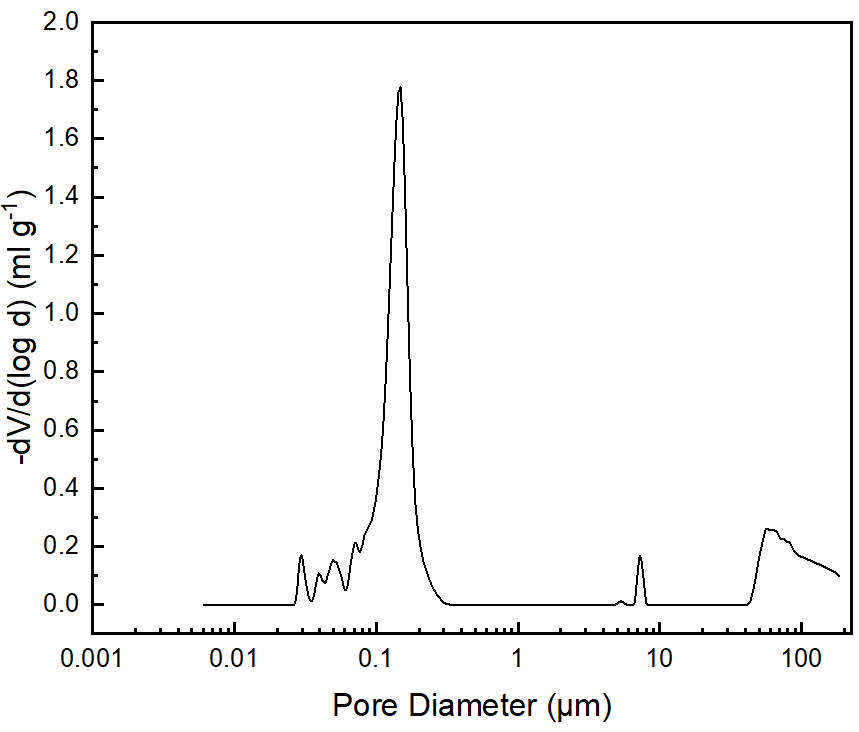

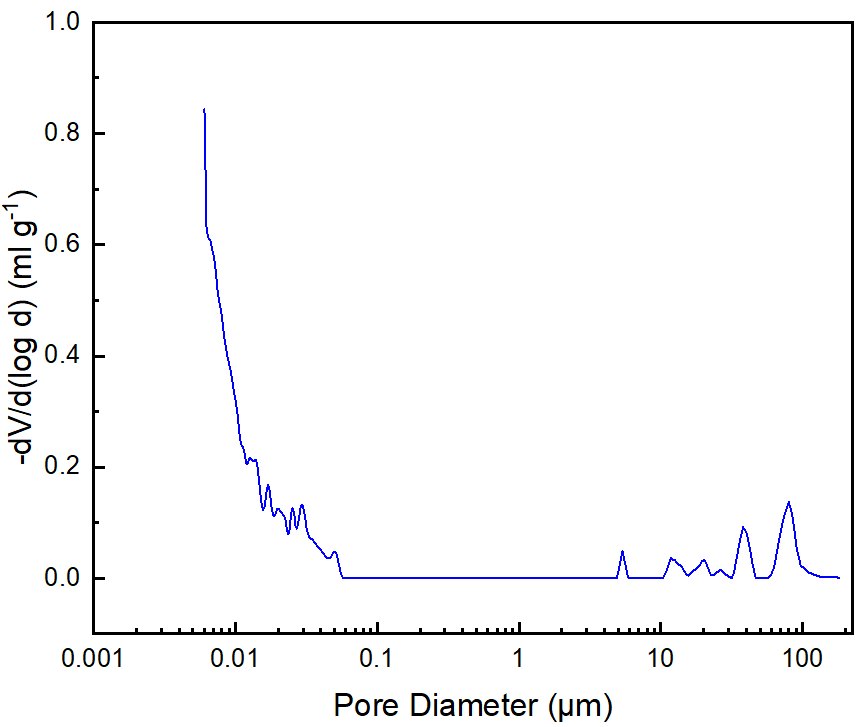

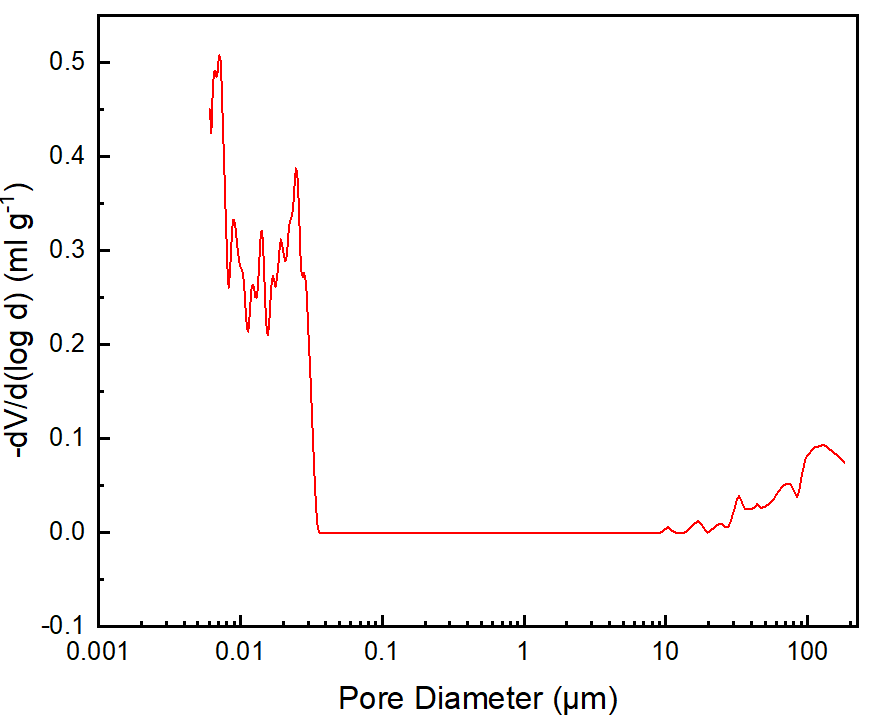

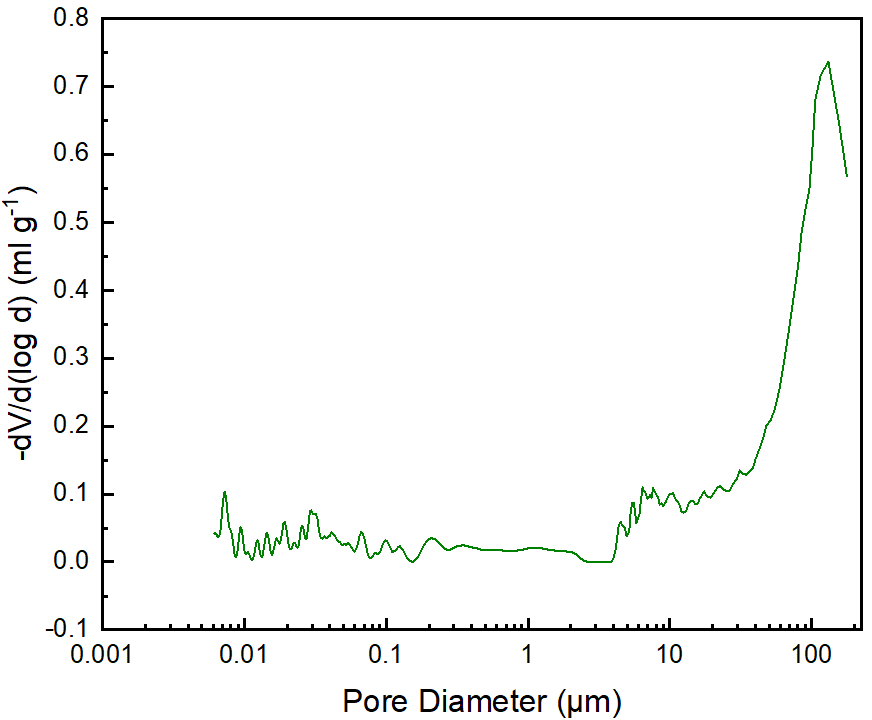


**(C)**

**(A)**

**(D)**

**(B)**

**Figure S27.** Meso- and macropore size distributions obtained by Hg porosimetry for _mono_ZIF-8 prepared in this work. (A) pZIF-8 (**─**), (B) mZIF-8-R (**─**), (C) mZIF-8-Δ (**─**), and (D) mZIF-8-CS (**─**).

# Dynamic Light Scattering

**Table S2.** Hydrodynamic diameter distribution (Peak) and cumulant (Z_avg_) particle size (nm) for aliquots from a range of reactions at 1-120 h.

| **Hours** | **Peak (d, nm)** | | | | |  | **Z_avg_ (d, nm)** | | | | |
| --- | --- | --- | --- | --- | --- | --- | --- | --- | --- | --- | --- |
|  | **_mono_ZIF-8** | **pZIF-8** | **mZIF-8-R** | **mZIF-8-Δ** | **mZIF-8-CS** |  | **_mono_ZIF-8** | **pZIF-8** | **mZIF-8-R** | **mZIF-8-Δ** | **mZIF-8-CS** |
| 1 | 396.4 | 663.5 | 70.5 | 89.3 | 95.4 |  | 1012.7 | 653.2 | 65.2 | 82.9 | 87.3 |
| 24 |  |  | 75.6 | 100.2 | 205.6 |  |  |  | 71.4 | 94.7 | 205.9 |
| 48 |  |  | 102.1 | 141.4 | 328.9 |  |  |  | 93.9 | 118.1 | 273.1 |
| 72 |  |  | 174.3 | 289.6 | 429.8 |  |  |  | 147.4 | 253.8 | 349.1 |
| 96 |  |  | 201.4 | 465.4 | 1156.1 |  |  |  | 152.9 | 409.6 | 567.7 |
| 120 |  |  | 513.4 |  | 1440 |  |  |  | 293.1 |  | 1336.7 |


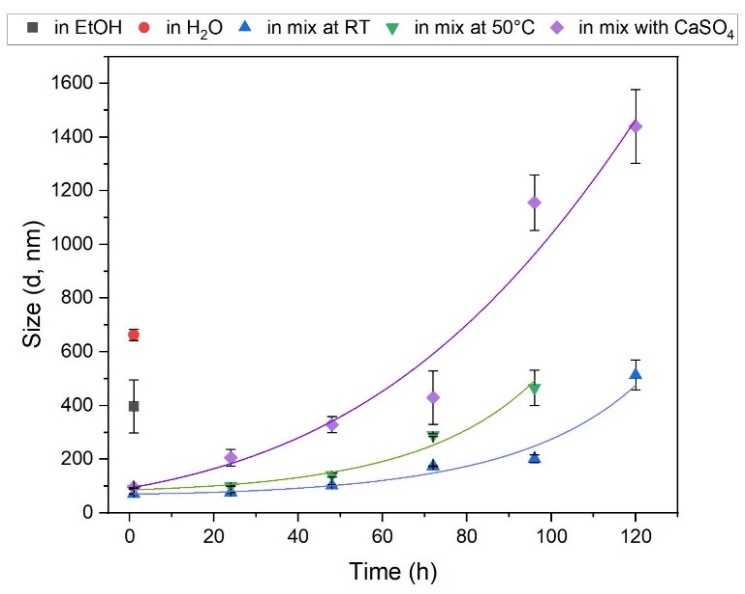

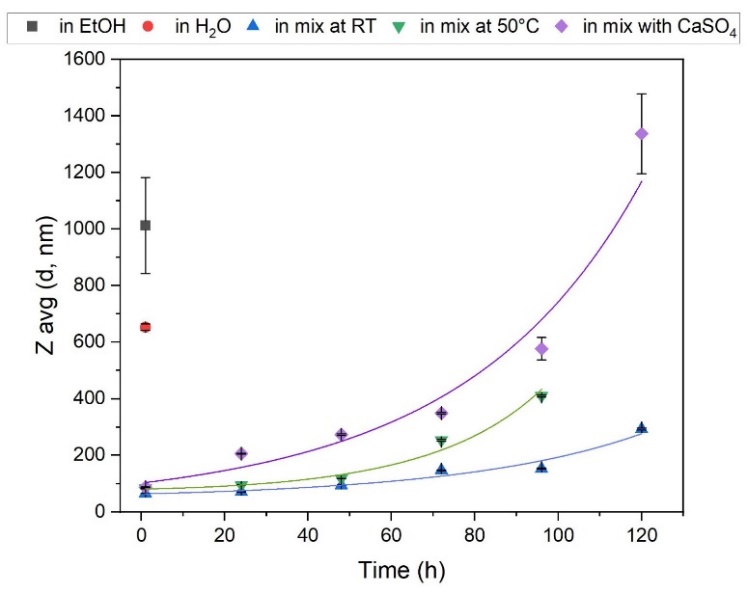


**Figure S28.** Graphical representations of hydrodynamic diameter distribution (Peak) and cumulant (Z_avg_) particle size (nm) against time (h) for aliquots from a range of reactions at 1-120 h. All readings are triplicated. ◼ EtOH. ⚫ H_2_O. ▲ EtOH−H_2_O, room temperature. ▼ EtOH−H_2_O, 50 ℃. ◆ EtOH−H_2_O, CaSO_4_.


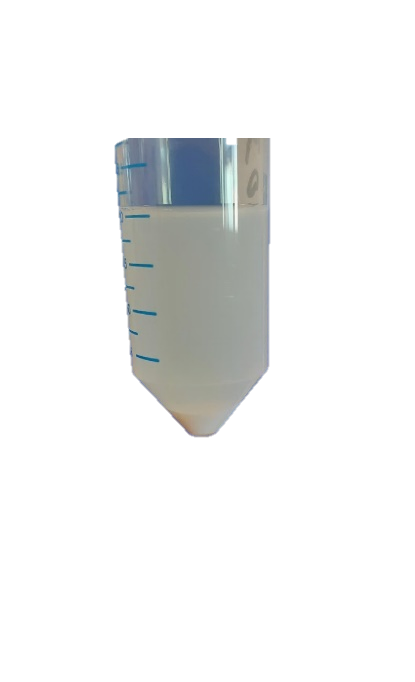

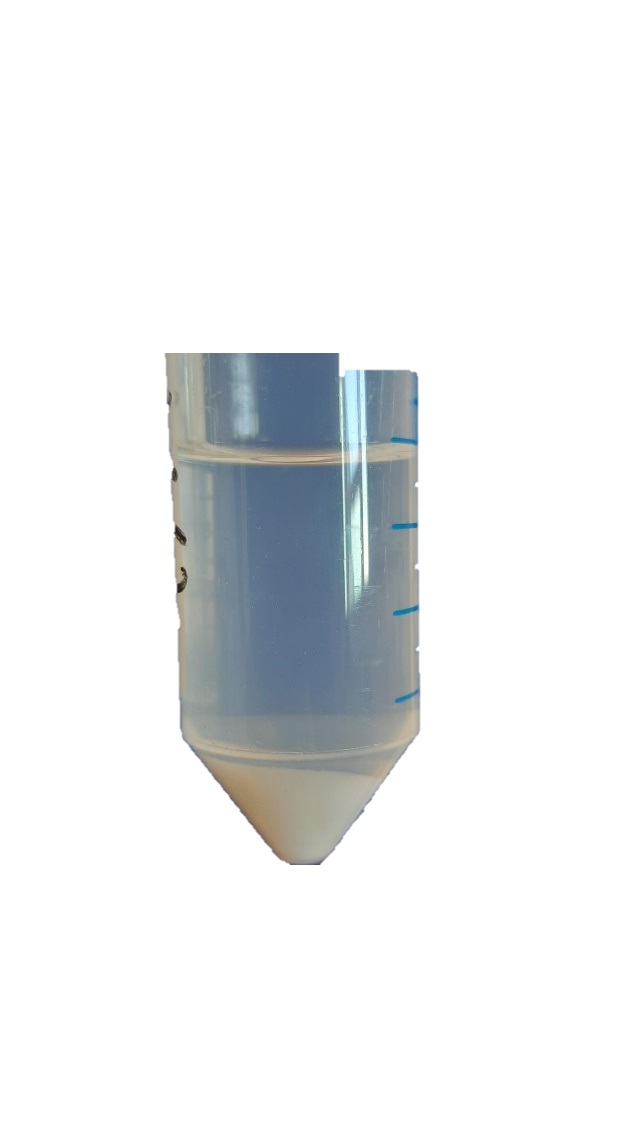


**Figure S29.** Samples of mZIF-8-R (120 h reaction time, left) and mZIF-8-Δ (96 h reaction time, right) after centrifugation at 5500 rpm for 20 minutes demonstrating the polydispersity of the former and the narrower dispersity of the latter.

# ζ-Potential

**Table S3.** ζ-Potential (mV) for aliquots from a range of reactions at 1-120 h. ◼ Rapid coagulation (ζ potential 0-5). ◼ Incipient instability (10-30). ◼ Moderate stability (30-40). ◼ Good stability (40-60). ◼ Excellent stability (>61).^4^ In EtOH, a value of 18 mV reflects rapid reaction with growing particles. In water, very large primary particles rapidly form and show moderate stability. In mixed-solvents, initial stability prevents collection of the colloid and leads to extended reactions. The subsequent trend in loss of stability matches with the observed reaction times needed to allow centrifugal isolation of colloid gel.

| **Hours** | **ζ-Potential (mV)** | | | | |
| --- | --- | --- | --- | --- | --- |
|  | **_mono_ZIF-8** | **pZIF-8** | **mZIF-8-R** | **mZIF-8-Δ** | **mZIF-8-CS** |
| **1** | 18.4 | 36.6 | 97.2 | 79.7 | 70.7 |
| **24** |  |  | 88.1 | 72.0 | 58.6 |
| **48** |  |  | 70.9 | 61.4 | 45.4 |
| **72** |  |  | 60.2 | 41.7 | 37.9 |
| **96** |  |  | 40.8 | 33.4 | 33.0 |
| **120** |  |  | 34.1 |  | 27.1 |


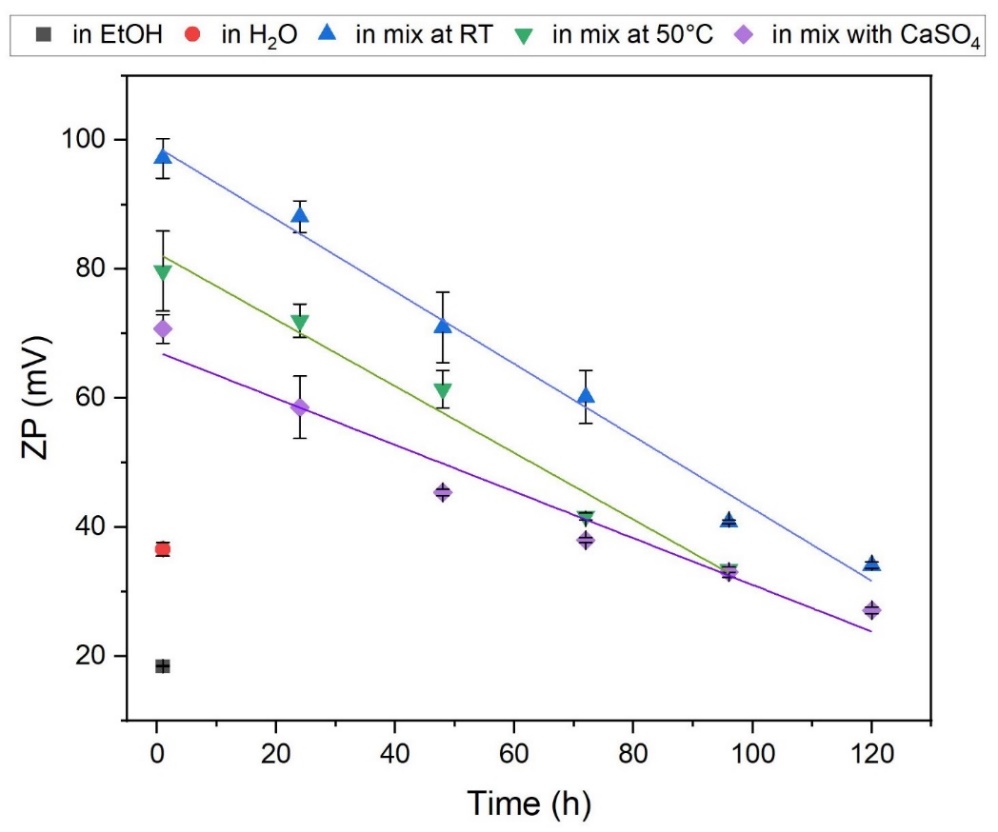


**Figure S30.** Graphical representations of ζ-potential (mV) against time (h) for aliquots from a range of reactions at 1-120 h. All readings are triplicated. ◼ EtOH. ⚫ H_2_O. ▲ EtOH−H_2_O, room temperature. ▼ EtOH−H_2_O, 50 ℃. ◆ EtOH−H_2_O, CaSO_4_.

# Solvent Density Measurements


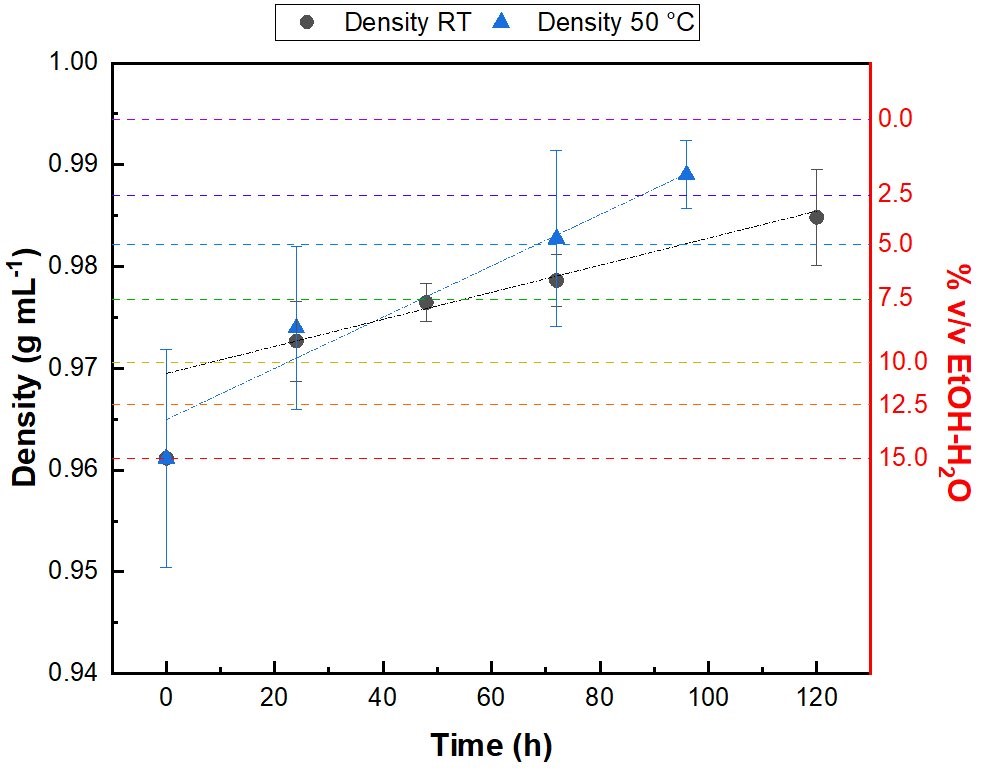


***Figure S31.*** *Calculated change in density of 15% v/v EtOH-H_2_O mixture (left y-axis), at RT (*●*) and at 50 °C (*▲*) versus time. Scattered points are fitted with linear trendlines. Calculated density of EtOH-H_2_O mixture at varied concentrations (right y-axis). All measurements were triplicated.*

# Post-synthetic doping


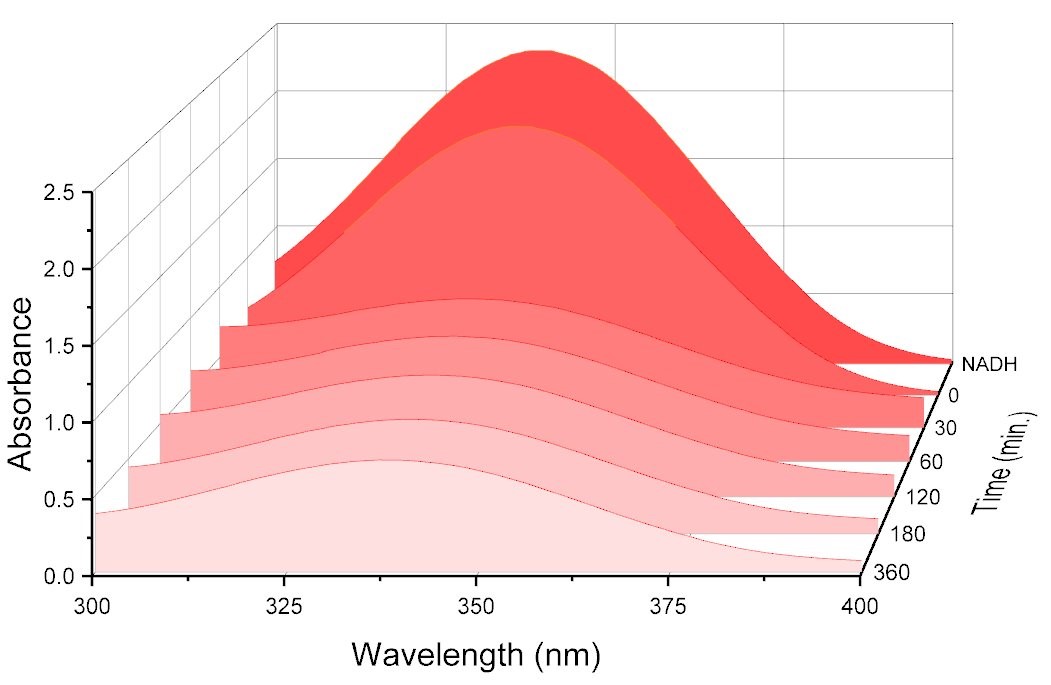


***Figure S32.*** *UV-Vis absorption spectra showing the results of post-synthetic doping of NADH (enzyme co-factor) in mesoporous mZIF-8-Δ. The elimination of NADH from the mother liquor is shown over 6 h (λ_max_ = 340 nm).^5^*

# References

1. Morris, W.*, et al.* NMR and X-ray Study Revealing the Rigidity of Zeolitic Imidazolate Frameworks. *The Journal of Physical Chemistry C* 116 (2012), 13307

2. Meng, X.*, et al.* Solid–Liquid Equilibrium of the Quaternary System Ca^2+^, Mg^2+^//SO_3_^2–^, SO_4_^2–^-H_2_O at 298.15 K. *Journal of Chemical & Engineering Data* 63 (2018), 4339

3. Kim, M.*, et al.* Bio-Templated Chiral Zeolitic Imidazolate Framework for Enantioselective Chemoresistive Sensing. *Angewandte Chemie International Edition* 62 (2023), e202305646

4. Kumar, A., and Dixit, C. K., 3 - Methods for characterization of nanoparticles. In *Advances in Nanomedicine for the Delivery of Therapeutic Nucleic Acids*, Nimesh, S.*, et al.*, (eds.) Woodhead Publishing(2017), pp 43

5. Osik, N. A.*, et al.* Nicotinamide adenine dinucleotide reduced (NADH) is a natural UV filter of certain bird lens. *Scientific Reports* 12 (2022), 16850
